# Endocrine Disruptors in Water and Their Effects on the Reproductive System

**DOI:** 10.3390/ijms21061929

**Published:** 2020-03-12

**Authors:** Andressa Gonsioroski, Vasiliki E. Mourikes, Jodi A. Flaws

**Affiliations:** Department of Comparative Biosciences, University of Illinois at Urbana-Champaign, Urbana, IL 61802, USA; avg5@illinois.edu (A.G.); mourike2@illinois.edu (V.E.M.)

**Keywords:** endocrine disruptors, water, reproduction

## Abstract

Anthropogenic contaminants in water can impose risks to reproductive health. Most of these compounds are known to be endocrine disrupting chemicals (EDCs). EDCs can impact the endocrine system and subsequently impair the development and fertility of non-human animals and humans. The source of chemical contamination in water is diverse, originating from byproducts formed during water disinfection processes, release from industry and livestock activity, or therapeutic drugs released into sewage. This review discusses the occurrence of EDCs in water such as disinfection byproducts, fluorinated compounds, bisphenol A, phthalates, pesticides, and estrogens, and it outlines their adverse reproductive effects in non-human animals and humans.

## 1. Introduction

Water safety and quality are fundamental to human development and well-being. Besides the pathogenic risk of microbes, several chemical contaminants present in water due to anthropogenic activities can impose risks to human and non-human animal health [1,2]. According to the United States Environmental Protection Agency (USEPA), the definition of contaminant is any physical, chemical, biological, or radiological substance or matter in water. Chemical contaminants are elements or compounds that can be naturally occurring or human-made [3]. The sources of chemical contamination in water are diverse. Chemicals can be present in water through the disinfection processes, chemical release in source water due to industry and livestock activity, and distribution from system components. Non-human animals and humans can be exposed to these compounds by ingesting, inhaling, or dermal contact with contaminated water. Some of the major chemicals that are known as water contaminants are endocrine disrupting chemicals such as disinfection byproducts, fluorinated substances, bisphenols, phthalates, pesticides, and natural and synthetic estrogens. Exposure to these compounds is associated with adverse health and reproductive outcomes in non-human animals and humans; thus, the presence of these chemicals in water has become a public health concern [4,5,6,7,8].

Studies have shown that the contaminants present in water can impair development, fertility, and reproductive function in non-human mammals, humans, and aquatic wild life. For instance, exposure to water disinfection byproducts in drinking water can cause cardiac anomalies in developing rat and porcine embryos [9,10]. Further, exposures to bisphenol A (BPA) and phthalates are known to reduce fertility in mammals by prematurely activating primordial follicles and altering levels of sex-steroid hormones [11,12,13,14,15]. Pesticides have been detected in drinking water sources, and some of these compounds are known reproductive toxicants. For example, exposure to some pesticides is associated with low sperm count and adverse pregnancy outcomes in non-human animals and humans [16,17,18]. Fluorinated substances also can be found in drinking water. Studies have reported that exposure to perfluorooctanoic acid (PFOA) and perfluorooctanesulfonic acid (PFOS) was responsible for impairing sperm viability and fecundability in non-human mammals and humans [19,20,21,22]. Moreover, water contaminated with synthetic estrogens can cause adverse pregnancy outcomes in non-human animals [23,24,25]. Collectively, these previous studies have shown that chemical contaminants in surface and drinking water worldwide can negatively influence the fertility and reproductive capacity of non-human animals and humans.

This review will discuss the occurrence of chemicals in water and their adverse reproductive effects in non-human mammals, humans, and aquatic life. Specifically, this review will focus on the following categories of chemicals found in water: disinfection byproducts, fluorinated compounds, BPA, phthalates, pesticides, and estrogens.

## 2. Water Disinfection Byproducts

The disinfection of drinking water was one of the most important public health achievements in the last century. The treatment of water with disinfectants such as chlorine substantially reduced the incidence of water-borne diseases, and it contributed to increases in life expectancy [26]. However, the reaction between disinfection agents and organic or inorganic matter in source water can form compounds called water disinfection byproducts (DBPs) [27]. The presence of DBPs in drinking water has become a human health concern because epidemiological studies have demonstrated associations between DBP exposure and an increased risk of cancer development and adverse reproductive outcomes [28,29,30,31,32].

### 2.1. Sources of Exposure to DBPs

Several factors can influence the formation of DBPs in drinking water. The presence of organic matter in source water plays a critical role in the formation of these compounds. Organic matter in water mostly consists of molecules such as fulvic, humic, carboxylic, and free amino acids, which are the primary precursors for formation of DBPs [33]. The chemical composition of source water is also an important factor regarding the formation of DBPs. For instance, in areas where the soil and source water are rich in bromine or iodide, the prevalence of brominated or iodinated DBPs tends to be higher than in areas lower in bromine or iodine [34,35]. Generally, increasing temperatures elevate the formation rates of DBPs. In addition, source water with low pH has been associated with high levels of DBPs because the most reactive form of chlorine, hypoclorous acid, is present in high concentrations in water sources with pHs lower than 7.5. Other important elements for DBP formation are the type and concentration of the disinfectant agent used to treat the water. For example, chlorine is known to have the highest potential to form DBPs, especially haloacetic acids, compared to chloramine, chlorine dioxide, or ozone [36].

A significant number of people are exposed to DBPs because of the widespread use of disinfectant agents to treat the water. The most common route of exposure is ingesting treated water, but other potential sources are consumption of food and beverages that were prepared with treated water [27]. Inhalation and dermal absorption also can occur by using showers, bath tubs, swimming pools, or steam rooms [27,36]. To date, more than 700 DBPs have been identified in drinking water [37]; however, only 11 of these compounds are regulated by the USEPA. 

The two major classes of DBPs are called trihalomethanes or total trihalomethanes (THMs or TTHMs) and haloacetic acids (HAAs). THMs were the first DBPs identified, and they are the most prevalent in drinking water [37]. Chloroform, bromoform, bromodichloromethane, and chlorodibromomethane are the four THMs that are currently regulated by the USEPA at the maximum contaminant level (MCL) of 0.080 mg/L [38] (Table 1). From 2013 to 2015, the average levels of TTHMs in US drinking water supplies were 0.03 mg/L [39]. HAAs are the second most prevalent DBPs in drinking water. In 1998, the USEPA first regulated the sum of five HAAs (bromoacetic acid, dibromoacetic acid, chloroacetic acid, dichloroacetic acid, and trichloroacetic acid), creating a group called HAA5. In 2016, the USEPA required monitoring for four additional HAAs, encompassing a group called HAA9. The MCL for HAA5 is 0.060 mg/L (Table 1), and levels in drinking water have been reported to be at or below this number [40].

DBPs have also been identified in swimming pool and spa water [41]. The water from these sources changes with the climate, the number and behavior of the users of pools or spas, activities of the swimmers, body fluids such as sweat and urine, as well as environmental contaminants brought into the pool on the skin (sun protectants, lotions) and clothes (bather load) [37,42]. All these components are suitable for reaction with disinfectant agents used to treat the water and can lead to the formation of DBPs. Daiber et al. reported that the total DBP concentrations are higher in water from pools and spas compared to their respective filling waters, which is likely due to the constant availability of disinfectants and organic matter input from swimmers [43]. Besides posing a risk to swimmers because of dermal absorption of DBPs, swimming pools and spas are a concern for public health because volatile DBPs can be trapped in the pool environment, especially in indoor pools, increasing the possible exposure to DBPs via inhalation [41].

### 2.2. Effects of DBPs on the Reproductive System

#### 2.2.1. Non-Human Animals

The toxicological effects of DBPs on developmental and reproductive outcomes have been studied in non-human animals from embryo development to birth. Teixido et al. investigated 10 regulated DBPs (four THMs, five HAAs, and bromate) to assess the developmental toxicity and genotoxicity of these compounds in zebrafish embryos. The authors reported that DBPs caused adverse developmental effects, significant reductions in the tail length (THMs exposure), and increases in malformation rates (tribromoacetic acid, dichloroacetic acid, and bromate exposure) [44]. In a different study, the developmental toxicity of 15 DBPs was assessed using the zebra fish embryo model. The toxicity rank order reported was: acetamides > HAAs > acetonitriles ~ nitrosamines. Furthermore, the study showed that brominated and iodinated DBPs tended to be more toxic than their chlorinated analogues [45]. Wang et al. tested the toxicity of halobenzoquinones, which are an emerging class of DBPs that have been detected in drinking water and swimming pool water [46]. They exposed zebrafish embryos to these compounds and compared the effects of halobenzoquinones to those found in zebrafish embryos exposed to HAAs. They showed that halobenzoquinones induced reactive oxygen species (ROS) generation and inhibited the antioxidative response of cells in developing zebrafish, resulting in death, physical malformations, oxidative DNA damage, and apoptosis. They also determined that the acute toxicity and ROS induction of halobenzoquinones was up to 200 times more potent than those induced by HAAs [46] (Table 2).

Besides causing developmental effects in zebrafish embryos, DBPs have been shown to be toxic to mouse, rat, and porcine embryos. In a study using CD-1 mouse embryos, the effects of exposure to different HAAs during a period of 24 h were assessed. Exposure to HAAs resulted in dysmorphogenesis, alterations in development of the neural tube and optic nerves, and abnormal heart development [47]. Andrews et al. exposed rat embryos to various concentrations of dichloro, dibromo, and bromochloroacetic acid (HAAs) for 48 h and then assessed dysmorphology. The primary effects of HAAs observed were dysmorphogenesis, heart defects, and to a lesser extent, prosencephalic, visceral arch, and eye defects. The developmental effect scores for embryos exposed to the combination of HAAs were higher when compared to the effect scores for embryos exposed to the single compounds, suggesting that the developmental toxicity of these DBPs was additive [9]. Further, exposure to environmentally relevant concentrations of bromodichloromethane, a type of THM, caused transcriptomic and epigenomic adaptive modifications compatible with the cardiac anomalies in porcine blastocysts [10] (Table 2). 

DBPs also have been shown to disrupt ovarian function, spermatogenesis, and fertility outcomes. To evaluate the effects of dibromoacetic acid on ovarian function, Bodensteiner et al. exposed female Dutch-belted rabbits daily to dibromoacetic acid through drinking water (0, 1, 5, or 50 mg DBA/kg body weight) from gestation day 15 throughout life [48]. They observed that dibromoacetic acid reduced the number of primordial follicles and total healthy follicles in prepubertal rabbits. In adult rabbits, dibromoacetic acid decreased the number of primordial follicles compared to the non-exposed rabbits [48]. In mice, iodoacetic acid inhibited antral follicle growth and reduced estradiol production by ovarian follicles in vitro [49]. To determine the mechanisms by which iodoacetic acid caused these alterations, Gonsioroski et al. [50] analyzed the gene expression and sex steroid hormone levels of mouse ovarian follicles in vitro. They showed that iodoacetic acid dysregulated the expression of apoptotic factors, cell cycle regulators, steroidogenic factors, and estrogen receptors, subsequently disrupting cell proliferation and steroidogenesis [50]. Narotsky et al. assessed the combined toxicity of regulated DBPs (TTHMs, HAAs, or TTHMs and HAAs) on the fertility indices of rats [51]. They observed that all three mixtures caused pregnancy loss and that HAAs alone or HAAs plus TTHMs increased resorption rates. In another study, the reproductive effects of an environmentally relevant mixture of DBPs representative of chlorinated drinking water were evaluated in rats in a multigenerational bioassay. The authors did not observe adverse effects of DBP exposure on pup weight, prenatal loss, pregnancy rate, gestation length, puberty onset in males, growth, estrous cycles, and hormone levels. However, the DBPs delayed puberty for F1 females, reduced caput epidydimal sperm counts in F1 adult males, and increased the incidence of thyroid follicular cell hypertrophy in adult females [52]. In male rats, dibromoacetic acid caused histopathologic changes in the testis and epididymis. Specifically, dibromoacetic acid caused the retention of spermatids, fusion of mature spermatids, and presence of atypical residual bodies in the epithelium and lumen of seminiferous tubules. In addition, the exposure caused distorted sperm heads, vacuolation of the Sertoli cell cytoplasm, vesiculation of the acrosomes of late spermatids, and marked atrophy of the seminiferous tubules [30]. Melnick et al. described similar testicular lesions in mice exposed to dibromoacetic acid. Specifically, lesions were characterized as spermatid retention and large atypical residual bodies in seminiferous tubules, which were suggested to be a result of the impaired degradative function in Sertoli cells [53] (Table 2).

#### 2.2.2. Humans

DBPs have been shown to be associated with adverse reproductive outcomes in women and men. For instance, in a retrospective cohort study conducted in Nova Scotia, Canada, consisting of 49,842 women who had a singleton birth between 1988 and 1995, exposure to chloroform and bromodichloromethane were associated with neural tube defects, cardiovascular defects, cleft defects, as well as chromosomal abnormalities [54]. For neural tube defects, the risk was increased with high exposure to bromodichloromethane but not chloroform. Further, a stronger relation between chloroform and chromosomal abnormalities was observed than between bromodichloromethane and chromosomal abnormalities [54]. In another study, Levallois et al. evaluated the association between maternal exposure to DBPs and the risk of delivering a small for-gestational-age neonate. HAA concentrations above the fourth quartile and THM or HAA concentrations above current water standards increased the risk for small for gestational age neonates [55]. In addition, in a study of 7438 singleton term babies in Bradford, England, TTHM exposure during pregnancy was associated with reduced birth weight [56]. Moreover, in a study of 2460 stillbirth cases from 1997 to 2004 in Massachusetts, chloroform and dichloroacetic acid exposures were associated with stillbirths [57]. In China, exposure to TTHMs was associated with decreased sperm concentration and serum testosterone in men [58]. Further, studies found that a GSTT1 polymorphism modified the association between exposure to bromo-THMs and decreased sperm motility. In addition, cytochrome P450 2E1 (CYP2E1) polymorphisms were associated with the internal blood concentrations of chloroform and TTHM [59] (Table 2). 

#### 2.2.3. Null Studies

Although some studies show that DBPs are associated with adverse reproductive outcomes, other studies have not found associations. For example, Cummings and Hedge did not observe effects of dibromoacetic acid in drinking water on the number of implantation sites found on gestational day 9, the number of pups per litter, the number of resorptions, or mean pup weight in rats [60]. Further, Weber et al. did not observe the effects of prenatal dibromoacetic acid exposure on daily sperm production, testicular sperm counts, epididymal sperm reserves, the morphology of seminiferous epithelium, or ovarian follicle counts in mice [61]. Narotsky et al. did not observe effects of a mixture of regulated DBPs on fertility, pregnancy maintenance, prenatal survival, postnatal survival, or birth weights in the parental, F1, and F2 generation of rats [62]. In human studies, no associations were found between exposure to DBPs and time to pregnancy, duration of gestation, small size for gestational age, stillbirths, preterm births, or birth weight [63,64,65,66,67,68]. Furthermore, some studies show that poor semen quality is not associated with exposure to DBPs in men [69,70,71]. 

These inconsistencies in the literature may be due to several factors. In experiments that use non-human animal models, the levels of DBP exposure are not always environmentally relevant, which can lead to discrepant findings. Further, the methods applied to treat non-human animals with DBPs do not always follow the routes of exposure for human and non-human animals (for example gavage versus drinking water). Thus, it is important for future studies to analyze the effects of single DBPs or mixtures of DBPs at environmentally relevant levels using relevant routes of exposure. In human epidemiological studies, differences in the size and genetic variability of the populations and variations in exposure levels makes comparison of results difficult among studies. The incorporation of subject behaviors into exposure evaluation, such as showering and swimming activities or the consumption of bottled or filtered water, could provide a better understanding of individual exposure to DBPs. Finally, few studies have been done on emerging DBPs and the underlying mechanisms of action of DBPs, opening up areas for additional research. 

## 3. Perfluoroalkyl and Polyfluoroalkyl Substances

Fluorinated substances are a wide group of organic and inorganic substances that contain at least one fluorine atom. A subset of these substances contains carbon atoms, on which all the hydrogen substituents have been replaced by fluorine atoms. These compounds are called perfluoroalkyl and polyfluoroalkyl substances (PFAS) [72]. In perfluoroalkyl substances, all carbons except the last one are attached to fluorines, and the last carbon attaches to the functional group. In polyfluoroalkyl substances, at least one, but not all carbons are attached to fluorines [73]. PFAS are human-made chemicals that have important properties such as hydrophobic and lipophobic nature, and chemical and biological stability. As a result of these properties, PFAS are used in a wide variety of consumer products and are highly persistent in the environment [74]. The presence of these chemicals in the environment is a concern for public health because exposure to PFAS has been associated with an increased incidence of tumors, endocrine disruption, impaired neurodevelopment, and adverse reproductive outcomes in humans and non-human animals [75,76,77,78,79,80,81,82,83,84].

### 3.1. Sources of Exposure to PFAS 

According to the USEPA, PFAS can be found in food packaged in PFAS-containing materials, processed with equipment that used PFAS, or grown in PFAS-contaminated soil or water. These compounds also can be found in commercial household products including stain- and water-repellent fabrics, nonstick products (pans), polishes, waxes, paints, cleaning products, and fire-fighting foams (a major source of groundwater contamination at airports and military bases where firefighting training occurs). Moreover, PFAS can be found in the workplace, including production facilities or industries that use these compounds. PFAS also can be found in drinking water, which is typically localized and associated with a specific facility (e.g., manufacturer, landfill, wastewater treatment plant, firefighter training facility). PFAS also can be present in living organisms, including fish, non-human mammals, and humans, where these chemicals have the ability to build up and persist over time [85]. Common PFAS are listed in Table 3.

As a result of the widespread use of PFAS, these chemicals can be found in surface and groundwater and subsequently in drinking water [86,87]. Studies have described the presence of PFAS in tap water in several countries, and the levels of these chemicals can vary largely depending on the location. In a study in Canada, average concentrations of PFOS and PFOA from the Great Lakes area were 3.4 ng/L and 1.8 ng/L, respectively, whereas samples from the rest of Canada had average concentrations of 0.4 and 0.7 ng/L, respectively [86]. In Brazil, the average levels of PFOS and PFOA in tap water were 6.7 ng/L and 2.7 ng/L, whereas in China, they were 3.9 ng/L and 10 ng/L, respectively [88,89]. In tap water samples from the United States (Ohio and Northern Kentucky), the average concentrations of PFOS and PFOA were 7.6 ng/L and 10 ng/L, respectively. PFAS have longer half-lives in humans than non-human animals, suggesting that humans could be more susceptible to PFAS toxicity than non-human animals [90,91,92]. To date, the USEPA does not have MCLs for PFAS in drinking waters, but this agency is analyzing the necessity of creating MCLs for PFOA and PFOS specifically [93].

### 3.2. Effects of PFAS on the Reproductive System

#### 3.2.1. Non-Human Animals

PFAS are known to disrupt reproductive function in non-human animals. Specifically, PFOA exposure damaged seminiferous tubules, increased spermatogonial apoptosis, and decreased testosterone levels in the testes of mice [19]. Exposure to PFOA decreased the number of mated and pregnant females per male mouse and disrupted blood testis barrier integrity [94]. Further, prenatal exposure to PFOA reduced the number of offspring, caused damage in the testes, disrupted reproductive hormones levels, and reduced expression of the *Dlk1-Dio3* imprinted cluster in testes in mice [95]. Prenatal exposure to PFOS decreased sperm count and serum testosterone concentration in male rat offspring [20]. Li et al. demonstrated that rats exposed to PFOS during puberty presented delayed Leydig cell maturation, decreased androgen production, reduced expression of cytochrome P450 11A1 (*Cyp11a1*), cytochrome P450 17A1 (*Cyp17a1*), and hydroxysteroid 17-Beta dehydrogenase 3 (*Hsd17b3*), and they disrupted the expression of apoptotic-related genes BCL2 associated X (*Bax*) and BCL2 apoptosis regulator (*Bcl-2*) in Leydig cells [96]. In female mice, PFOA exposure caused a delayed or absence of vaginal opening, lack of estrous cycling, decreased ovarian levels of steroidogenic acute regulatory protein (STAR), CYP11A1, 3-Beta dehydrogenase 1 (HSD3B1), and HSD17B1, and reduced protein levels of amphiregulin and hepatocyte growth factor in the mammary glands [97]. In mice, Chen et al. showed that maternal exposure to PFOA inhibited corpus luteum function, decreased levels of serum progesterone, decreased the ovarian expression of *Star, Cyp11a1*, and *Hsd3b1*, increased the ovarian expression of tumor protein (*p53)* and *Bax*, and reduced the expression of *Bcl-2* in the ovary, leading to embryo resorption, reduced fetal growth, and reduced postnatal survival [98]. Furthermore, PFOA exposure induced apoptosis and necrosis in mouse oocytes, which is likely related to reactive oxygen species (ROS) generation and gap junction intercellular communication disruption between the oocyte and the granulosa cells [99]. Working with female rats, Du et al. found that neonatal and juvenile exposure to PFOA or PFOS dysregulated the hypothalamic–pituitary–gonadal (HPG) axis, leading to advanced puberty onset, increased levels of serum luteinizing hormone and estradiol, and the reduced expression of kisspeptin 1 (*Kiss1)*, kisspeptin 1 receptor (*Kiss1r)*, and estrogen receptor alpha (*Esr1)* in the hypothalamic anteroventral periventricular and arcuate nuclei [81] (Table 4).

#### 3.2.2. Humans

PFAS have been associated with reproductive and fertility dysfunction in men and women. In an epidemiologic study in Denmark, men with high combined semen levels of PFOS and PFOA had decreased normal sperm numbers compared to men with low semen levels of PFOS and PFOA [100]. In another study in Denmark, in utero exposure to PFOA was associated with lower sperm concentration and total sperm count in adult men [101]. Further, in vitro exposure to PFOA impaired human sperm penetration in synthetic mucus, which was likely caused by excessive ROS production, compromising human sperm penetration ability and acrosome reaction by canceling progesterone-induced Ca^2+^ signaling [102]. In addition, men exposed to PFOA for up to 2 h exhibited altered sperm motility due to plasma-membrane disruption [21]. In China, maternal exposure to PFAS was associated with shorter anogenital distance in boys, providing evidence that PFAS may function as EDCs to affect male genital development [110]. Moreover, PFOA and PFOS exposure were associated with reduced semen quality, testicular volume, penile length, and anogenital distance in men in the Veneto region, Italy. This same study demonstrated that PFOA plays an antagonistic role on the binding of testosterone to androgen receptor, possibly dysregulating the HPG axis [112] (Table 4). 

In women, exposure to PFAS has been associated with endometriosis in the US and China [103,104,105]. Further, levels of PFAS in blood have been associated with decreased serum levels of estradiol, progesterone, sex hormone-binding globulin, follicle-stimulating hormone (FSH), and testosterone [106,107]. Plasma concentrations of PFAS in pregnant women in the Danish National Birth Cohort were associated with low birth weight and long time to pregnancy [22,108]. Moreover, the maternal–infant research on environmental chemicals study, a cohort study of pregnant women across Canada, showed that plasma levels of PFOA and PFHxS were associated with reduced fecundability [109]. In Swedish women, prenatal exposure to PFOA was associated with higher odds for small for gestational age [113]. In a recent study, PFAS exposure was associated with increased age at menarche and irregular menstrual periods in young women. The same study reported a significant alteration in the expression of genes related to embryo implantation in Ishikawa cells exposed to PFOA compared to non-exposed cells [111] (Table 4). 

Although several studies have shown that PFAS exposure causes adverse reproductive and health effects, little is known about emerging PFAS and their effects on the environment and human health. For example, perfluoro-2-propoxypropanoic acid (PFECA), a PFOA replacement known as “GenX”, has been shown to have higher toxicity than PFOA when correcting for differences in toxicokinetics. However, the effects of “GenX” on reproductive outcomes are unclear [114]. In addition, few studies have examined the effects of exposure to a mixture of PFAS on non-human animal and human health, which could provide more information about the potential interactions between individual PFAS. Future studies should include these factors to improve our understanding of PFAS toxicity and adverse health outcomes.

## 4. Bisphenol A

Bisphenol A (BPA) is an important compound in the bisphenol (bishydroxyarylalkanes) group [115]. Currently, BPA is a high production volume chemical that is widely used in manufacturing polycarbonate plastics and epoxy resins for industrial use [116]. Polycarbonate plastics are used in food and drink packaging (water and infant bottles, compact discs, impact-resistant safety equipment, medical devices), whereas epoxy resins are used as lacquers to coat metal products (food cans, bottle tops, water supply pipes) [117]. Human exposure to BPA is a public health concern because BPA has the ability to bind membrane and nuclear receptors such as androgen, estrogen, and thyroid receptors, causing endocrine disruption, tumors, adverse reproductive outcomes, and transgenerational effects [118,119,120,121,122]. 

### 4.1. Sources of Exposure to BPA

The primary source of exposure to BPA is diet, but BPA is ubiquitous in the environment, air, dust, and water. BPA can leach into food from the protective internal epoxy resin coatings of canned foods and from consumer products such as polycarbonate tableware, food storage containers, water bottles, and baby bottles [117]. Canada was the first country to prohibit the sale and importation of BPA-containing baby bottles [123]. Several states in the US banned the use of BPA in cups, bottles, thermoses, baby food and infant formula containers, or thermal paper [124]. Further, the French National Assembly and Senate suspended the use of BPA in all applications that have contact with food [125]. In contrast, the European Food Safety Authority concluded that BPA was not a threat for the health of consumers of any age. In addition, the United States Food and Drug Administration (USFDA) declared that BPA is safe at the current levels occurring in foods [126]. Although controversies about BPA regulation exist, studies have shown that this chemical is an endocrine disruptor, which means that this compound is able to trigger adverse health effects at low and environmentally relevant doses [127,128].

BPA is ubiquitous in aquatic environments and can be detected in rivers, effluent from sewage treatment plants, and water from water treatment plants [129,130]. Specifically, the mean concentrations of BPA in the Huangpu River in China were 22.93 ng/L in surface waters, 84.11 ng/g in suspended solids, and 7.13 ng/g dry weight in surface sediments [130]. Further, a study in Taiwan determined that BPA concentrations in drinking water were increased with contact time in polyvinyl chloride (PVC) pipes [131]. In some provinces of South Africa, BPA was found to be present in 62% of the analyzed drinking water and wastewater samples [132]. Further, in raw water and tap water samples in France, BPA levels were up to 1430 ng/L and between 9 and 50 ng/L, respectively [133]. In wastewater treatment plants, BPA was found at concentrations of 60.5 ng/L in five states in India, 1960 ng/L in 49 samples from Xiamen City in China, and 412 ng/L in one sample from Dalian City, China [134,135,136]. The USEPA reported that BPA concentrations in US drinking water are typically below 1 µg/L [137]. Although exposure to BPA through tap water is a minor source of human BPA exposure, bottled mineral water may also lead to exposure [138]. 

### 4.2. Effects of BPA on the Reproductive System

#### 4.2.1. Non-Human Animals

BPA is known to cause adverse reproductive outcomes in non-human animals. Specifically, it has been demonstrated that BPA disrupts the HPG axis in mice, rats, and zebrafish [139,140,141,142,143,144]. In mice, studies have shown that BPA exposure reduced sperm motility, reduced normal sperm morphology, decreased sperm membrane integrity, decreased sperm count, impaired sperm function, induced spermatocyte apoptosis, and impacted testicular development [145,146,147,148,149]. In females, BPA exposure is known to cause altered mammary gland development and morphology. Specifically, in utero exposure to BPA resulted in altered development, increased epithelial volume, and the altered ductal morphology of mammary glands in mice [121,150]. Further, Ibrahim et al. showed that adult BPA exposure increased the number of the ducts and acini of the mammary gland, with hyperplasia in their lining epithelium in rats [151]. These studies agree that mammary gland changes due to BPA could lead eventually to an increased incidence of mammary gland cancer [119] (Table 5). 

Exposure to BPA has been shown to affect the ovaries. Prenatal BPA exposure inhibited germ cell nest breakdown in ovaries of the F1 generation in mice, decreased the numbers of primordial, primary, preantral, and total healthy follicle numbers at post-natal day 21, and decreased estradiol levels in female rats dosed for 1 year, suggesting that BPA targets the ovary [11,12]. Further, BPA exposure initiated an excessive premature activation of primordial follicles in mouse mature ovaries via the phosphatase and tensin homolog/phosphatidylinositol-3-kinase/ protein kinase B (PTEN/PI3K/AKT) signaling pathway by downregulating phosphatase and tensin homolog (PTEN) expression in vivo [152] (Table 5).

Other studies showed that BPA exposure affected uterine function by impairing implantation and the establishment of pregnancy in mice and rats, caused intra-uterine growth restriction in mouse fetuses, altered steroid hormone signaling in mouse uteri, and impaired the number of nerve fibers in the wall of the porcine uterus [13,153,154,155,169]. Collectively, these studies show that BPA exposure affects the reproductive tract and reduces non-human animal fertility (Table 5).

#### 4.2.2. Humans

BPA has been shown to be associated with impaired reproductive function in men and women. For example, in a study in Greece, very high concentrations of plasma BPA were associated with azoospermia in men [156]. Further, in a cross-sectional study with young men, high levels of urinary BPA were positively associated with serum luteinizing hormone (LH) levels and negatively associated with sperm concentration [5]. Other studies show that BPA urinary levels are associated with decreased sperm concentration, reduced semen quality, decreased antioxidant levels, reduced sperm DNA integrity, decreased motility, and an increased percentage of immature sperm [157,158,159]. Moreover, BPA levels in urine were inversely associated with both the number of oocytes retrieved in women undergoing in vitro fertilization and the serum levels of estradiol [160]. Lee et al. observed associations among high urinary BPA levels and increased serum levels of testosterone, estradiol, and pregnenolone in girls diagnosed with precocious puberty [161]. In a recent study, serum BPA concentrations were higher in women diagnosed with polycystic ovary syndrome compared to women in the healthy groups [170]. Moreover, increased serum BPA concentrations were associated with reduced fecundability among women without preconception folic acid supplementation. Wang et al. also observed decreased fecundability in Chinese women attempting pregnancy with high concentrations of BPA in the urine [162]. Further, studies have shown that high serum and urinary BPA levels were associated with increased miscarriage risk [163,164,171]. In addition, high levels of BPA in maternal blood, urine, or amniotic fluid were associated with decreased gain weight during pregnancy and low birth weight [165,166,167,168]. Collectively, these studies indicate that BPA exposure is negatively associated with adverse reproductive function in men and women (Table 5). 

Substantial evidence demonstrates that BPA is a reproductive toxicant in non-human animals and humans. For that reason, structural analogues have been used as BPA alternatives, but some of these compounds have been also identified as toxicants [172]. Thus, future research is necessary to elucidate the mechanisms by which BPA analogues act in the reproductive system. 

## 5. Phthalates

Phthalates are versatile plasticizers, lubricants, and solvents, which are used in a number of industries. They can be grouped into high and low molecular weight categories based on their chemical functional groups and carbon number. High molecular weight phthalates are commonly found in food storage containers, including single and reusable water bottles, children’s toys, PVC products such as construction materials and clothing, and medical equipment such as intravenous tubing and transfusion bags. The high molecular weight phthalates discussed in this review are di (2-ethylhexyl) phthalate (DEHP), benzyl butyl phthalate (BBP), di-isononyl phthalate (DiNP), and di-n-octyl phthalate (DnOP). Low molecular weight phthalates are ubiquitously used in cosmetics and personal care products, pharmaceuticals, and adhesives. The low molecular weight phthalates discussed in this review are diethyl phthalate (DEP), dimethyl phthalate (DMP), dibutyl phthalate (DBP), and diisobutyl phthalate (DiBP). 

### 5.1. Sources of Exposure to Phthalates

Since phthalates participate in non-covalent interactions with plastic polymers, they readily leach out, contaminating both the individuals exposed to them and the environment [173]. In comparison, low molecular weight phthalates are more water soluble than long-chain high molecular weight phthalates and thus, they are more likely to accumulate in finished drinking water and drinking water sources [174]. Furthermore, phthalates are relatively stable in the environment, which leads to environmental buildup. Even phthalates that do not directly contaminate drinking water sources can enter these sources from buildup in sediment, agricultural soil, and urban soil. Global meta-analyses of phthalates found contamination at concentrations of 0.01–115 mg/kg in sediment, 0.02–264 mg/kg in agricultural soils, and 0.01–30.1 mg/kg in urban soils [173]. Both high and low molecular weight phthalates enter drinking water through two primary routes: (1) phthalates from industrial runoff contaminate drinking water sources such as surface and groundwater and (2) phthalates can leach into our drinking water from plastic food and water storage units such as water bottles.

In 2006, raw drinking water in California was tested for phthalate contamination. DBP (1.44 µg/L and 8.34 µg/L) and DEHP (2.67–5.94 µg/L) were the leading phthalate contaminants followed by DMP (0.08–0.789 µg/L), DEP (0.899–1.49 µg/L), and BBP (0.053–1.19 µg/L) [175]. In a nationwide survey of six phthalates in drinking water sources across China, DEP, DMP, DMP, BBP, DEHP and DnOP were all detected. DBP and DEHP were found most abundantly, with median values of 0.18 µg/L [176]. More recently, DBP concentrations in the Yangtze River in the Delta City of China have exceeded the Chinese surface water standards [177]. In addition to parent phthalate compounds, bioactive phthalate metabolites named phthalate monoesters have recently been identified in drinking water sources from 24 Chinese cities. Monomethyl phthalate (MMP), monoethyl phthalate (MEP), monoisobutyl phthalate (MiBP), mono-n-butyl phthalate (MnBP), and mono-2-ethylhexyl phthalate (MEHP) were detected at mean concentrations of 12.1 ± 18.0, 2.4 ± 5.8, 11.3 ± 37.2, 36.3 ± 103, and 9.9 ± 18.0ng/L, respectively [178]. 

Most single-use plastic water bottles are made of polyethylene terephthalate (PET). They are popular around the world due to their convenience and low cost. High and low molecular weight phthalates leach out of PET bottles and into the drinking water. The storage of these bottles at various temperatures can accelerate the leaching of phthalates into the water, increasing exposure levels. Drinking water from the bottles of 10 popular PET brands stored at various temperatures was tested for the presence of phthalates in Beijing, China. DEP, DMP, and DBP were found in all samples at concentrations ranging from 101.97 to 709.87 µg/kg. DEP, DMP, DBP, BBP, DOP, and DEHP were detected at levels between 0.18 and 0.71 µg/L in water from bottles stored at room temperature. The concentrations of these same six phthalates increased from 0.19 to 0.98 µg/L when the bottles were stored at outdoor temperatures above 24 °C. DBP was the main phthalate component that increased in response to higher storage temperatures [179]. DEHP was the most commonly detected phthalate in bottled water in Greece, with a median concentration of 350 ng/L. DNP (44 ng/L) and DEP (33 ng/L) were also detected in bottled water, although at lower concentrations than DEHP [180]. DnBP (0.06–6.5 µg/L), DIBP (0.1–1.89 µg/L), and DEHP (0.02–0.16 µg/L) were detected in Portuguese drinking water stored in both PET and glass bottles. DEHP was detected in water from PET bottles at concentrations up to five times higher than water from glass bottles. However, DnBP was higher in the water stored in glass bottles, and it reached an average concentration of 6.5 µg/L [181]. The leaching capacity of various phthalates from drinking containers exposed to sunlight and baby feeders subject to disinfection by boiling, autoclaving, and oven disinfection yielded 10 detectable phthalates and identified that DMP, DMEP, DEP, and DBP had the greatest leaking potential, with average concentrations between 9 and 112.5 µg/L. 

### 5.2. Effects of Phthalates on the Reproductive System

#### 5.2.1. Non-Human Animals

DEHP has been shown to cause early reproductive senescence in male CD-1 mice by impairing testosterone production, reducing sperm quality, and decreasing fertility [6]. DBP has been shown to decrease the numbers of sperm and Sertoli cells in the F1, F2, and F3 generations of male SpragueDawley rats born to exposed females [182]. In utero DEHP exposure has also been shown to delay the onset of puberty in the F3 generation and decrease sperm count, while increasing the frequency of abnormal seminiferous tubules in the F3 and F4 generations of male CD-1 mice [183] (Table 6).

In laboratory animal studies, phthalate exposure has been associated with a decline in female reproductive health. Experimental data from laboratory animals show that phthalate exposure is associated with increased resorptions and decreased pregnancy, implantations, and fetal weights of offspring [184,203]. Female mice exposed to MEHP in utero (corn oil, 100, 500, or 1000 mg/kg of MEHP) exhibited premature reproductive senescence compared to female mice that received only corn oil [185]. F1, F2, and F3 female mice exposed to DEHP in utero (0, 0.05, 5 mg/kg/day of DEHP) had reduced oocyte quality and reduced embryonic developmental competence compared to non-exposed female mice. Genes responsible for ovarian and embryonic development were also dysregulated in exposed mice [186]. In utero exposure to DEHP resulted in F1 female offspring with reduced estrogen levels at proestrus, increased FSH levels at both proestrus and estrus, and significantly decreased thecal cell layers compared to control mice. The F3 generation of females from this same study had decreased weights overall, decreased rates of pregnancy, and increased litter size compared to control [187]. In another study on the transgenerational effects of DEHP exposure, the onset of puberty was accelerated and estrous cyclicity was disrupted in all three generations of female mice. DEHP exposure decreased the rate of pregnancy and fertility indices in the F1 generation. DEHP exposure also increased female-biased litters and decreased anogenital distance in the F3 generation compared to control [188] (Table 6). 

DEHP exposure also accelerated folliculogenesis in adult mice orally exposed to DEHP as well as its metabolite MEHP. Following DEHP exposure, the mice had decreased primordial follicle numbers and increased primary, preantral, or antral follicle numbers compared to non-exposed mice [185,204]. DEHP exposure also has been associated with the transgenerational dysregulation of folliculogenesis in female mice. F1 female mice exposed to DEHP in utero had decreased antral follicle numbers at PND 21, and by PND 60, they had decreased primary and preantral follicle numbers compared to control. The exposed females had significantly fewer germ cells as well as accelerated folliculogenesis compared to control [188]. 

In male laboratory animals, phthalates interfere with sex hormone steroidogenesis in reproductive organs via alterations in steroidogenic gene transcription [205]. HSD3B, the steroidogenic enzyme that catalyzes the conversion of 3β-hydroxysteroids into 3-keto-steroids, has been identified as a phthalate target in testicular tissue [206] (Table 6). 

Parental phthalate compounds and their bioactive monoester metabolites have also been found to interfere with female sex hormone steroidogenesis in female reproductive tissues via transcriptional dysregulation. Female mice administered DEHP at 5, 10, and 15 days postpartum had a significant decrease in the expression of genes responsible for androgen synthesis in the theca cells [189]. These genes included luteinizing hormone/choriogonadotropin receptor *(Lhcgr)*, *Cyp17a1*, *Star*, and low-density lipoprotein receptor (*Ldlr*). Ovaries collected from treated mice had significantly decreased concentrations of progesterone, 17beta-estradiol, and androstenedione in their ovaries and reduced LH in serum compared to mice that did not receive the treatment. The thecal nuclear envelope was also deformed in the follicles of exposed mice compared to control [189]. In a transgenerational study of DEHP exposures, F1 generation female mice prenatally exposed to DEHP had significantly decreased serum testosterone levels and increased serum 17beta-estradiol levels compared to the F1 generation of non-treated mice. The F2 generation from this same study had significantly decreased serum progesterone levels compared to the F2 generation of non-treated mice [188] (Table 6).

#### 5.2.2. Humans

Phthalates are recognized as reproductive toxicants with endocrine disrupting capabilities in both males and females. Epidemiological studies have identified associations between maternal urine phthalate and phthalate metabolite levels and anogenital distance in boys. Although DEHP and DBP have strong associations with reduced anogenital distance in boys, the metabolites of DiNP, which is a popular DEHP replacement, have moderate associations with reduced anogenital distance [190,191,207]. Inverse associations have been reported between urinary concentrations of DEHP and DiNP metabolites and sperm concentration, sperm motility, and testosterone levels [192,193,194,195,196]. DBP and BBP urinary metabolites also have inverse associations with sperm concentration and sperm motility [176,192,194,197,198]. Data from the National Health and Nutrition Examination Survey (NHANES) 2011–2012 in the US yielded inverse associations between increasing DiBP metabolite concentrations in urinary phthalates and decreased testosterone [208]. Similar associations were found in a study of infertile men in Taiwan and in fertile and infertile Chinese men [196,199]. Increased DEHP exposure also has been associated with the increased apoptosis of sperm cells and the increased generation of ROS in sperm cells, whereas DEHP, DBP, and BBP have all been associated with increased sperm aneuploidy [195,209,210,211] (Table 6). 

In utero exposure to MEP and MBzP was associated with increased testosterone levels in girls aged 8–13 in a Mexico City birth cohort [200]. Urinary levels of MEHP and MBzP in 8-year-old girls from Taiwan were positively associated with increased serum progesterone levels, and urinary levels of MBzP and MBP were positively associated with increased serum FSH levels [201]. Chronic occupational phthalate exposure in women has been associated with decreased pregnancy rates and increased miscarriage rates [212]. Phthalate exposure also has been associated with complications including anemia, toxemia, and preeclampsia in pregnant women [213]. In a study of midlife women, urinary phthalate metabolite levels were associated with increased risk of ever experiencing hot flashes, having had hot flashes within 30 days of sample collection, and more frequent hot flashes [202]. Collectively, these data show that phthalate exposure is associated with adverse reproductive outcomes throughout a woman’s lifetime (Table 6). 

Since different types of phthalates are used by the industry and they are relatively stable in the environment, future studies should focus on the effects of exposure to mixtures of phthalates. These mixtures should represent the environmental relevance of phthalates specific to a given area; thus, it may be possible to elucidate if there are synergistic effects through different phthalates exposure. 

## 6. Pesticides

Prior to the 1940s, elements such as arsenic, mercury, copper, and lead were used in pest management. Due to low water solubility, their accumulation in water was not a concern. Synthetic organic pesticides were introduced during World War II, and since then, the United States Geological Survey (USGS) estimates that 1 billion pounds of pesticides are applied annually to agricultural land, non-crop land, and urban areas in the United States. The use of these synthetic organic compounds has allowed the US to become the largest producer of food in the world and kept lethal vector-borne diseases such as malaria at bay. However, these accomplishments may have come at a significant cost to human health. Drinking water, which is sourced from either groundwater or surface water, is a potent vehicle of exposure to pesticides for both humans and non-human animals. The Netherlands National Institute of Public Health and Environmental Protection concluded that “groundwater is threatened by pesticides in all European states” [214]. Groundwater is especially vulnerable to persistent pesticide contamination because, in contrast to flowing bodies of water, it remains still. Pesticide contamination of these sources, in addition to the intentional application of pesticides for water disinfection, results in chronic exposure to nontarget species and a significant risk of adverse reproductive health outcomes.

### 6.1. Sources of Exposure to Pesticides

Pesticides are intentionally applied to various water systems to combat disease-causing and intrusive organisms. They are added to waterways such as canals, rivers, lakes, and streams to control mosquitoes, weeds, and invasive fish. Disinfectant pesticides are used in water treatment plants to remove bacterial and viral contamination from drinking water. They are also applied to water used in large-scale irrigation systems, especially in humid and tropical environments. This is done to protect crops from infestations, as well as humans from contracting vector-borne diseases such as malaria. A larger proportion of pesticide contamination in water systems is not due to intentional application. According to the USEPA, pesticides applied to farms, gardens, and lawns run off into both ground and surface water systems that feed drinking water supplies in both agricultural and urban settings [215]. Furthermore, the likelihood of a pesticide reaching drinking water is significantly greater when it is spilled, dumped, or misused in comparison to labeled uses. Point sources of pesticide contamination include pesticide manufacturing plants, mixing-and-loading facilities, spills, waste disposal sites, sewage treatment plants, and wastewater recharge facilities such as wells and basins. During the recharge of groundwater, pesticides can seep into and through the soil ending up in aquifers. Nonpoint sources of pesticide contamination in water are the dominant source of pesticides found in both ground and drinking water because they are diffuse and widely dispersed. They include runoff to streams from agricultural and urban land, seepage to groundwater in areas where pesticides are heavily used, leaching out of paint on ships, and illegal disposal by homeowners down the drain. When rain falls on a treated area, it can carry pesticides to surface water sources very far from their point of application. Some pesticides are even capable of moving in the air from their point of application to very distant surface water reservoirs. Atrazine and two of its metabolites were detected in 50% of precipitation and 23% of particulate phase atmospheric samples taken from Lake Michigan, US. Furthermore, the atrazine concentrations in the precipitation were not reflective of local land use, insinuating long-range transport through the atmosphere [216]. 

Pesticides and their metabolites have been detected in drinking water sources across the world. In some cases, their levels exceed the regulatory limits of their respective countries. Atrazine, along with its metabolite desethlatrazine, and simazine were the most frequently detected pesticides above regulatory levels across Europe [217]. Acetochlor was first registered for use in the US in 1994. By 1995, it was detected in multiple sources groundwater around the US, exemplifying how rapidly these chemicals can accumulate in aqueous environments [218]. Pesticides remain in drinking water sources for a considerable time, whether they are applied intentionally or by runoff. This is, in part, due to their chemical properties such as adsorption and solubility. For example, atrazine has low adsorption into soil particles and readily leaches into water. Compared to other herbicides, atrazine has relatively higher solubility [216], making it an ideal candidate for accumulation in drinking water sources. Neonicotinoids, a relatively new family of insecticides, are synthetic nicotine derivates as well as very small and water-soluble compounds [219]. In turn, this leads to neonicotinoids having high leaching and runoff potential [220]. According to the manufacturer, only 5% of applied imidacloprid, a widely popular neonicotinoid, is spread throughout the crop, and the rest dissipates into the environment [221]. The chemical characteristics and evidence of environmental runoff make neonicotinoids ideal candidates for persistence in drinking water sources [220]. Less soluble pesticides such as dichlorodiphenyltrichloroethane (DDT) and chlordane can adhere to sediment and can consequently persist in waterways for years [222]. Although DDT was banned in the US and many other countries around the world in the early 1970s, DDT and its metabolites are still being detected in drinking water sources globally [223,224]. 

Herbicides are the greatest offenders of pesticide contamination in drinking water sources in the United States, Europe, and Asia [225]. Researchers and government agencies alike have detected levels of popular herbicides in finished drinking water and in drinking water sources. Namely, atrazine, simazine, metolachlor, and acetochlor are the most commonly detected herbicides in drinking water and drinking water sources. 

After herbicides, insecticides are the second most commonly detected pesticide contaminant in drinking water and drinking water sources. Appreciable concentrations of neonicotinoid insecticides, as well as their organophosphate predecessors have been detected in many countries around the world. Organophosphates are recognized as the most acutely toxic family of pesticides to nontarget species, including humans [226]. They can be transported in water long distances from their source of application to surface and groundwater reservoirs. They have been detected in snow, fog, and rainwater [227,228]. Furthermore, their primary route of groundwater degradation is hydrolysis, meaning that water contaminated with organophosphate parent compounds is also likely contaminated with hydrolytic metabolites, some of which are even more toxic than their associated parent compounds [229,230]. 

Prechlorination is used by all water treatment facilities for water disinfection and odor control. The prechlorination of organophosphates in water significantly increases the concentration of transformed oxons as the primary byproduct of organophosphate oxidation in finished drinking water. The organophosphate oxons are more toxic than their parent compounds, and they are more water soluble, making them even more difficult to remove from the finished drinking water. The organophosphate insecticides most commonly detected in drinking water and drinking water sources are diazinon, chlorpyriphos, and malathion. Diazinon and malathion were detected in Ethiopian drinking water from wells, springs, and tap at concentrations ranging from 1.6 to 5.7 μg/L and 7.3 to 14 μg/L, respectively [231]. Chlorpyriphos ethyl, a chlorpyriphos metabolite, was the most frequently detected insecticide in surface water in Greece, and its average concentration (0.031 μg/L) exceeds the EU environmental quality standard [232]. Malathion was detected in 25% of drinking water aqueducts in Venezuela (2.03 μg/L). Diazinon was found at levels as high as 26.31 μg/L in these Venezuelan aqueducts. Although these levels are not beyond the Venezuelan limits, they far exceed US and EU EPA levels [233]. In the US, malathion was reported at levels up to 0.18 μg/L in surface water [234]. 

### 6.2. Effects of Atrazine on the Reproductive System

Atrazine is the most common surface and groundwater herbicide contaminant worldwide. It was detected in 84% of drinking water samples in Croatia (5–68 ng/L), and in 74.1% of drinking water sources in the Guangxi province of China [235,236]. Surface and tap water from Northern Italy also is contaminated with atrazine (5 ng/L) and atrazine-desethyl, which is an atrazine metabolite (11 ng/L) [237]. Atrazine has been detected ubiquitously in Lake Michigan, US open water samples, atmospheric samples, and in 11 tributaries that flow into the lake [216].

#### 6.2.1. Non-Human Animals

Atrazine has been shown to cause reproductive toxicity in animal models. Song et al. identified numerous toxic effects of atrazine on the reproductive system of male rats, including irregular and disordered arrangement of seminiferous epithelium, decreased numbers of spermatozoa, increased numbers of abnomal spermatozoa, decreased levels of total antioxidant capacity, decreased serum levels of testosterone and inhibin-B, and increased serum levels of FSH and LH [238]. Female rats exposed to atrazine were found to have significantly delayed vaginal opening, reduced ovary, uterine, and pubertal body weights, and dysregulated estrous cycles with extended periods of diestrous [239,240] (Table 7). 

#### 6.2.2. Humans

Several epidemiological studies have shown associations between atrazine exposure and adult reproductive outcomes in people. In a study of fertile men in agricultural Missouri in the US, men exposed to atrazine, alachlor, and diazinon had 40% lower sperm counts compared to men in US urban areas. After controlling for potential confounding factors, the highest correlation was found between reduced sperm count and high concentrations of atrazine in urine [253]. In a comparison of 13 communities receiving drinking water with elevated levels of atrazine to nearby communities receiving water from other sources, levels of atrazine, metolachlor, and cyanazine were each significant predictors of age-adjusted community rates of intrauterine growth retardation. This association was strongest for atrazine [254]. The mechanisms by which atrazine causes toxicity likely stem from it being an endocrine disrupting chemical that mediates reproductive abnormalities through targeting the HPG axis [255] (Table 7).

### 6.3. Effects of Simazine on the Reproductive System

Simazine is the second most commonly detected triazene herbicide in surface and groundwater and is commonly used in urban areas [241]. Simazine (16 ng/L) was detected in all surface and tap water tested in Northern Italy [237]. 

#### Non-Human Animals

Several studies indicate that simazine causes reproductive toxicity in animal models. Female offspring of mice exposed to environmentally relevant doses of simazine had shortened anogenital distance, decreased whole body, ovarian, and uterine weights, and increased apoptotic granulosa cells in the ovaries compared to control [241]. Female Wistar rats exposed to simazine for 21 days experienced delayed vaginal opening, decreased numbers of normal cycles, late onset of first estrus, and decreased prolactin compared to control [242]. Male offspring exposed to simazine in utero had decreased body, testicular, and epididymal weights, increased testicular apoptosis, and decreased sperm concentrations compared to control [243]. Leydig cells exposed to atrazine and simazine had dose-dependent increases in progesterone and testosterone compared to controls. These endpoints were mediated by the induction of *Star, Hsd3b6, Hsd17b3*, and the downregulation of *Hsd3b1, Cyp17a1,* and *Srd5a1*, which changed in a dose-dependent manner [256] (Table 7).

### 6.4. Effects of Metolachlor on the Reproductive System

Along with atrazine, metolachlor is one of the most consistently detected herbicides in the world. Environmental monitoring studies in the US have detected appreciable concentrations of metolachlor in drinking water sources in North Carolina (17–5866 ng/L), Georgia (0.09–10.5 μg/L), and 53% of drinking water sources in California in the US [257,258,259]. Metolachlor has been detected in 54% of the drinking water in Zegrab Croatia, more than 50% of the water sources sampled in La Rioja Spain, and 31.4% of samples tested in the Guangxi province of China [235,236,260]. 

#### 6.4.1. Non-Human Animals

Prepubertal Wistar rats exposed to metolachlor had increased serum concentrations of testosterone, estradiol, and FSH, a reduction in dihydrotestosterone (DHT), and no change in LH compared to control. Metolachlor-treated male rats also had increased fluid in their seminal vesicles, early onset of puberty, and morphological abnormalities of seminiferous epithelium compared to control [244] (Table 7).

#### 6.4.2. Humans

Metolachlor contamination in drinking water was found to be a significant predictor of intrauterine growth retardation in Iowa, US communities when compared to neighboring communities whose water was not contaminated with metolachlor [254] (Table 7).

### 6.5. Effects of Acetochlor on the Reproductive System

Similar to the herbicides mentioned above, acetochlor has been identified as a leading water contaminant globally. This herbicide has been detected in 66.9% of the China’s source and drinking water, with an average concentration of 33.9 ng/L [261]. More recently, 32% of drinking water in Croatia (107–117 ng/L) was found to be contaminated with acetochlor [235]. 

#### Non-Human Animals

Acetochlor has been recognized as an endocrine disrupting chemical by the USEPA and European Environmental Agency. Further, male mice exposed to acetochlor had significantly increased epididymal weight compared to control. Testicular tissues of exposed mice also had decreased superoxide dismutase and glutathione activity and increased malondialdehyde activity, which can collectively lead to increased ROS in the testis [245]. Neonatal exposure to acetochlor has been shown to alter pubertal development in female rats by accelerating vaginal opening and altering estrous cyclicity [246] (Table 7).

### 6.6. Effects of Organophosphates on the Reproductive System

#### Non-Human Animals

Several studies have shown that organophosphates are reproductive toxicants. Malathion was found to significantly lower sperm motility and quantity and to increase rates of sperm malformation in rats. Treatment with malathion also disrupted expression of apoptotic factors in the testes of rats by downregulating *Bcl-2*, which is an anti-apoptotic factor, and upregulating *Bax*, which is a pro-apoptotic factor [247]. Adult male rats exposed to chlorpyriphos were found to have decreased sperm count, sperm viability, and sperm motility as well as increased DNA damage in sperm cells. They also had arrested spermatogenesis, negative tubular differentiation and repopulation indexes, and decreased Leydig cell numbers compared to controls [248]. Another study found that male rats treated with chlorpyriphos exposure had significantly increased serum LH and decreased serum testosterone levels [249] (Table 7).

### 6.7. Effects of Neocotinoides on the Reproductive System

Neonicotinoids, a relatively new class of insecticides, pose a unique threat to drinking water supplies. Their unifying property as synthetic neonicotinoid derivatives makes them very small molecules that are water soluble, thus increasing the likelihood that they will leach out of soils and remain in drinking water sources for quite some time. Researchers in central China found neonicotinoids in all raw, finished, and tap water samples originating from the Han River and Yangtzee River in central China. The median sum concentration of all the neonicotinoids in these samples was 27.7 ng/L, with a range of 13.4–186 ng/L [262]. Clothianidin, imidacloprid, and thiamethoxam have been detected in finished drinking water in the Midwest US, with concentrations ranging from 0.24 ng/L to 57.3 ng/L [263]. Their presence in finished drinking water globally exemplifies their persistence during conventional water treatment as well as the elevated risk of exposure to humans and non-human animals (Table 7). 

#### Non-Human Animals

Despite being a new class of insecticides, evidence already exists that neonicotinoids are reproductive toxicants. In developing male rats, treatment with imidacloprid resulted in decreased sperm concentration, increased apoptosis and seminal DNA fragmentation, decreased serum testosterone, and decreased glutathione in the testis [250]. Adult females exposed to imidacloprid had decreased ovarian weight, increased FSH, and decreased LH and progesterone levels in serum, increased lactoperoxidase activity in the ovary, and decreased antioxidant capabilities in the ovary [251]. Adult male rats exposed to clothianidin had significantly decreased epididymal weights and elevated palmitic, linoleic, and arachidonic acids in their testes [252]. Male mice exposed to clothianidin experienced these same pathologies, and they also exhibited decreased glutathione peroxidase immunoreactivity. Despite their growing popularity and hydrophilic chemical composition, regulations are not in place for neonicotinoid contamination in drinking water (Table 7). 

Since one of the sources of exposure to pesticides is ingestion of water and food, future studies should focus on the cumulative effects of different pesticides on the endocrine and reproductive systems. In addition, studies on the underlying mechanisms of toxicity of pesticides are crucial for understanding the toxic effects in non-human animals and humans and for developing appropriate strategies to reduce risk of pesticide toxicity.

## 7. Estrogens

Natural and synthetic estrogens are contaminants in the environment [264,265]. These compounds are endocrine disruptors that can alter gonadal steroid signaling by interacting with estrogen receptors [266]. The most prevalent estrogens found in the environment are estrone, 17β-estradiol, 17α-estradiol, and estriol. These estrogens are naturally produced by humans and non-human animals [267]. Natural phytoestrogens from plants are also released in the environment [268]. Environmental contamination with estrogens has become a public health concern because of the ability of these compounds to disrupt the endocrine system, impair reproductive function, and trigger adverse health effects [269]. 

### 7.1. Sources of Exposure to Estrogens

The sources of estrogens in the environment can be diverse. The cattle industry is one major source of estrogens released into the environment, especially because the industry uses growth-regulating steroids to enhance cattle growth rates [265]. Further, estrogens have been detected in solid waste and effluents from livestock and agricultural areas [270]. Moreover, water has been polluted with estrogens released from sewage plants. The human source of estrogens is mainly through urine excretion. For example, pregnant women excrete between 260 and 790 μg/day of estrone, 280 to 600 μg/day of 17β-estradiol, and 6000 and 10,000 μg/day of estriol [265]. Although ethinylestradiol from birth control pills is an additional endocrine disrupting chemical that contributes to the feminization of aquatic species, the contribution of this compound to drinking water estrogenicity has been shown to be less than that from other sources [271]. This could be because the only source of ethinylestradiol in drinking water is assumed to be therapeutic use and this compound transforms to estrone under all but nitrate-reducing conditions [272,273].

Estrogens are found in rivers, wastewater, and drinking water. For instance, estrone was the most commonly detected estrogen in water samples derived from streams associated with livestock operations in 12 states in the US [274]. Liu et al. estimated that the amount of estrogens from livestock (56.8 g·d^−1^) released into water environments was nearly two-fold higher than from humans (35.2 g·d^−1^) in Shangai [275]. Further, estriol was present in the highest average concentrations (summer: 3.6 ng/L; winter: 2.7 ng/L) followed by 17α-estradiol in analyzed water samples from the Hanjiang River in China [276]. In addition, ethinylestradiol and estriol were the main estrogens responsible for the estrogenic potencies in samples of source and drinking water in eastern China. Moreover, a study revealed the existence of 17β-estradiol and ethinylestradiol in water samples collected from Meiliang Bay, China. Esteban et al. detected estriol 3-sulfate, estrone, and its metabolite at frequencies of 14% and 29% in analyzed samples of sewage treatment plants in the Madrid region [277].

### 7.2. Effects of Environmental Estrogens on Reproduction

#### 7.2.1. Non-Human Animals

Exposure to estrogens present in the environment is known to impair development and reproductive function. For example, a study conducted in northwestern Ontario, Canada showed that chronic exposure to low levels of ethynylestradiol led to the feminization of male fishes through the production of vitellogenin mRNA and protein, impacts on gonadal development, and altered oogenesis in fish [278]. Further, exposure to estrogens present in effluents and in downstream waters caused feminized male fishes to have elevated concentrations of the egg yolk protein precursor (vitellogenin), decreased testes size, loss of secondary sex characteristics, and intersex [279,280,281,282]. In addition, Huang et al. observed strong estrogenic effects associated with the concentration of estrogens (estrone, 17β-estradiol, and diethylstilbestrol) in mosquitofish in China [283]. Exposure to ethynylestradiol affected metamorphosis and altered sex ratios in frogs during vulnerable periods of development [284] (Table 8).

Mice and rats also have been shown to be sensitive to environmental estrogens. Specifically, Derouiche et al. showed that 17α-ethinylestradiol-exposed mice males and their progeny expressed increased sexual behavior in a dose-dependent manner [23]. In a recent study, Meyer et al. demonstrated that prenatal exposure to low doses of 17α-ethinylestradiol (environmentally relevant concentrations) impaired remodeling of the spiral arteries, increased the weight of the placenta, and increased the number of pups large for gestational age in mice [24]. In addition, prenatal exposure to 17α-ethinylestradiol caused high abortion rates and modifications of maternal behavior in rats [25]. Prepubertal exposure to 17α-ethinylestradiol advanced puberty, increased kisspeptin signaling to GnRH neurons, and increased *Gnrh* expression [285].

#### 7.2.2. Humans

Exposure to exogenous estrogens has been associated with an increased risk of breast cancer in women in Spain [286]. Moreover, urinary phytoestrogens levels were associated with idiopathic infertility in men in China [287]. Together, these studies show that estrogens that contaminate surface waters worldwide can negatively influence the fertility and reproductive capacity of non-human animals and humans (Table 8).

Besides the information available about relationships between estrogens and adverse reproductive outcomes, data are limited on the levels and types of estrogens in the environment. In addition, the role of estrogen contamination in different ecosystems and populations is still not well understood. Future investigations should be conducted to fill these gaps in knowledge.

## 8. Conclusions

Growing evidence indicates that anthropogenic contaminants are present in water across the world and that they can impose negative health effects in non-human animals and humans. These environmental toxicants can act directly or indirectly on the reproductive system, impairing development and fertility. Considering that the routes of exposure to these chemicals are not restricted to the ingestion of water, the levels of exposure for some of these compounds can be much higher than those from water alone. Further studies in a wide variety of populations and species are required to explore the long-term consequences of exposure to contaminants present in water and their reproductive effects. Although the effects of chemicals among species may differ, non-human animal models serve as a basis for scientific experimentation as they provide mechanistic, effectiveness, and toxicological information about EDCs. Additionally, it is necessary to consider the effects of mixtures of contaminants from different categories to mimic the normal environmental exposure in domestic animals, wild life, and humans. More studies are needed in a variety of populations to determine if the impacts of environmental chemicals on reproduction differ by populations in different locations worldwide.

## Figures and Tables

**Table 1:** USEPA Drinking Water Regulations for DBPs.

Disinfection Byproduct	MCLG ^1^ (mg/L) ^4^	MCL ^2^ or TT ^3^ (mg/L) ^4^
Bromate	0	0.010
Chlorite	0.8	1.0
Haloacetic acids (HAA5)	n/a	0.060
Dichloroacetic acid	0 mg/L	
Trichloroacetic acid	0.02 mg/L	
Monochloroacetic acid	0.07mg/L	
Bromoacetic acid	n/a	
Dibromoacetic acid	n/a	
Total Trihalomethanes (TTHMs)	n/a	0.080
Bromodichloromethane	0 mg/L	
Bromoform	0 mg/L	
Dibromochloromethane	0.06 mg/L	
Chloroform	0.07 mg/L	

^1^ Maximum Contaminant Level Goal (MCLG)-The level of a contaminant in drinking water below which there is no known or expected risk to health. MCLGs allow for a margin of safety and are non-enforceable public health goals. ^2^ Maximum Contaminant Level (MCL)-The highest level of a contaminant that is allowed in drinking water. MCLs are set as close to MCLGs as feasible using the best available treatment technology and taking cost into consideration. MCLs are enforceable standards. ^3^ Treatment Technique (TT)-A required process intended to reduce the level of a contaminant in drinking water.^4^ Units are in milligrams per liter (mg/L). Milligrams per liter are equivalent to parts per million (PPM).Source: USEPA, 2010 [38].

**Table 2:** Effects of DBPs on the Reproductive System.

Chemical	Exposure Window	Dose	Model/Study Population	Effects	Conclusions	Reference
Trihalomethanes (THM4), haloacetic acids(HAA5), bromate	Developmental exposure	20–100 μg/mL	Zebra fish embryos	Adverse developmental effects, reduced tail length, increased malformation rates	Weak capacity of the selected disinfection products to cause developmental effects at environmentally relevant concentrations.	[44]
Chloroacetamide, bromoacetamide, iodoacetamide, chloroacetic acid, bromoacetic acid, iodoacetic acid, chloroacetonitrile, dichloroacetonitrile, trichloroacetonitrile, bromoacetonitrile, dibromoacetonitrile, iodoacetonitrile, n-nitrosodimethylamine, n-nitrosodiphenylamine, n-nitrosomorpholine	Developmental exposure	1–500 µM	Zebra fish embryos	Yolk sac and pericardial edema Axis, eye, snout, jaw, somite, pectoral fin, and caudal fin malformations Delayed developmental progression, reduced sensitivity to touch	The selected DBPs altered zebra fish development.	[45]
2,6-dichloro-1,4-benzoquinone, 2,5-dichloro-1,4-benzoquinone, 2,5-dibromo-1,4-benzoquinone, tetrachloro-1,4-benzoquinone, tetrabromo-1,4-benzoquinone, dichloroacetic acid, dibromoacetic acid, iodoacetic acid	Developmental exposure	0–16 µM	Zebra fish embryos	Increased mortality, reactive oxygen species, DNA damage, apoptosis, uninflated swim bladder, tail injury, pericardial edema, shortened body length, shortened yolk sac extension, developmental delay	Halobenzoquinones are acutely toxic, causing oxidative damage and developmental toxicity to zebrafish larvae.	[46]
Trichloroacetic acid, dichloroacetic acid, chloroacetic acid, bromoacetic acid, tribromoacetic acid, tri- fluoroacetic acid, difluoroacetic acid, dibromoacetic acid	Developmental exposure	1 to 17,000 µM	CD-1 mouse embryos	Prosencephalic hypoplasia, non-closure, impaired optic development, malpositioned and/or hypoplastic pharyn- geal arches, and perturbation of heart development	The selected haloacetic acids analyzed are potential developmental toxicants.	[47]
Dibromoacetic acid	Gestational, lactational, and adult exposure	0, 1, 5, or 50 mg/kg	Female Dutch-belted rabbits	Reduction in number of primordial follicles and total healthy follicles In adult animals, fewer primordial follicles	Chronic exposure to dibromoacetic acid diminishes the ovarian primordial follicle population.	[48]
Chloroacetic acid, bromoacetic acid, iodoacetic acid	48 and 96 h in vitro exposure of ovarian follicles	0.25–1.00 mM of chloroacetic acid; 2–15 µM of bromoacetic acid or iodoacetic acid	Ovarian follicles from CD-1 mice	Inhibition of antral follicle growth Reduction of estradiol levels	The selected monoHAAs inhibit the growth of antral follicles and reduce estradiol levels compared to controls in a dose-response manner.	[49]
Iodoacetic acid	96 h in vitro exposure of ovarian follicles	2–15 µM of iodoacetic acid	Ovarian follicles from CD-1 mice	Inhibition of antral follicle growth, reduction of estradiol levels Altered expression of genes related to the cell cycle, ovarian steroidogenesis, apoptosis, and estrogen receptors Altered levels of steroid hormones	Iodoacetic acid exposure inhibits follicle growth, decreases cell proliferation, and alters steroidogenesis in mouse ovarian follicles in vitro.	[50]
Chloroform, bromodichloromethane, chlorodibromomethane, bromoform chloroacetic acid, dichloroacetic acid, trichloroacetic acid, bromoacetic acid, dibromoacetic acid	Gestational exposure	1–72 mg/kg body weight	F344 rats	Increased pregnancy loss, embryo resorption, eye malformations (anophthalmia, microphthalmia)	Haloacetic acids cause pregnancy loss and contribute to the potency of the THM-HAA mixture in causing pregnancy loss.	[51]
106 DBPs and other chemicals identified or measured in a chlorinated concentrate water	Gestational, lactation, prepubertal exposure	N/A	Sprague−Dawley rats	Delayed puberty for F1 females Reduced caput epidydimal sperm counts in F1 adult males Increased incidence of thyroid follicular cell hypertrophy in adult females	Exposure to DBPs affects puberty, sperm production, and thyroid cells.	[52]
Dibromoacetic acid	Adult exposure	0, 125, 250, 500, 1,000, and 2,000 mg/L in the 2-week and 3-month studies, and 0, 50, 500, and 1,000 mg/L in the 2-year studies	F344/N rats and B6C3F_1_ mice	Delayed spermiation and atypical residual bodies in male rats and mice Atrophy of the germinal epithelium in rats	Dibromoacetic acid adversely affects male reproductive tissues/processes.	[53]
Chloroform, bromodichloromethane	Gestational exposure	Levels in the water-distribution systems: Chloroform: <50 µg/L, 50–74 µg/L, 75–99 µg/L, and 100 µg/L, and bromodichloromethane: <5 µg/L, 5–9 µg/L, 10–19 µg/L, and >20 µg/L	49,842 women who had a singleton birth in Nova Scotia, Canada between 1988 and 1995	Increased risk of neural tube defects Increased risk of chromosomal abnormalities	Chloroform and bromodichloromethane gestational exposure is associated with increased risk of neural tube defects and chromosomal abnormalities.	[54]
Trihalomethanes and haloacetic acids	Gestational exposure	Concentrations of trihalomethanes and haloacetic acids in the water-distribution systems (0.1–49.3 µg/L)	Pregnant women aged 25 to 34 years. Term newborn cases with birth weights <10th percentile (n = 571) were compared with 1925 term controls with birth weights ≥10th percentile. Québec City, Canada area	Increased risk of small for gestational age	Trihalomethane and haloacetic acid gestational exposure is associated with increased risk of small for gestational age.	[55]
Trihalomethanes and haloacetic acids	Gestational exposure	Maternal DBPs exposures (0.2–45.6 µg/L)	Longitudinal multi-ethnic birth cohort study in Bradford, England with pregnant women	Birth weight reduction of approximately 50 g	Exposure to trihalomethane during pregnancy is associated with adverse fetal growth, including reduced birth weight.	[56]
Chloroform, bromodichloromethane, dibromochloromethane, bromoform, trichloroacetic acid, dichloroacetic acid, monobromoacetic acid and summary DBP measures (trihalomethanes, haloacetic acids, brominated trihalomethanes, and DBP9 (sum of trihalomethanes, haloacetic acids)	Gestational exposure	Second-trimester disinfectant byproduct (µg/L) exposure levels for cases and controls, 1998–2004 (0–31.9 µg/L)	2460 stillbirth cases 1997–2004 in Massachusetts, US	Positive associations between stillbirth and DBP exposure	Trihalomethanes exposure increases risk of stillbirth.	[57]
Trihalomethanes	Adult exposure	Baseline blood concentrations of trihalomethanes (mean of 0.58–57.68 ng/L)	401 men in Wuhan, China between April 2011 and May 2012	Moderate levels of bromodichloromethane were associated with decreased sperm count and declined sperm linearity	Elevated trihalomethane exposure may lead to decreased sperm concentration and serum total testosterone.	[58]
Trihalomethanes	Adult exposure	Baseline blood concentrations of trihalomethanes (mean of 0.58–57.68 ng/L)	401 men in Wuhan, China between April 2011 and May 2012 and	Genetic polymorphisms of CYP2E1 and GSTZ1 were associated with semen quality	A combination of genetic susceptibility and environmental exposure to trihalomethanes may be associated with semen quality parameters.	[59]

**Table 3:** Common PFAS.

Abbreviation	Chemical Name
PFOS	Perfluorooctane sulfonic acid
PFOA (aka C8)	Perfluorooctanoic acid
PFNA	Perfluorononanoic acid
PFDA	Perfluorodecanoic acid
PFOSA (aka FOSA)	Perfluorooctane sulfonaminde
MeFOSAA (aka Me-PFOSA-AcOH)	2-(N-Methyl-perfluorooctane sulfonamido) acetic acid
Et-FOSAA (aka Et-PFOSA-AcOH)	2-(N-Ethyl-perfluorooctane sulfonamido) acetic acid
PFHxS	Perfluorohexane sulfonic acid

Source: ATSDR, 2017 [73].

**Table 4:** Effects of PFAS on the Reproductive System.

Chemical	Exposure Window	Dose	Model/Study Population	Effects	Conclusion	Reference
PFOA	Adult exposure	0, 0.31, 1.25, 5, and 20 mg/kg/day by oral gavage for 28 days	BALB/c male mice	Damaged the seminiferous tubules Reduced testosterone and progesterone levels in the testis in a dose-dependent manner Reduced sperm quality and altered expression of 93 proteins	PFOA exposure can impair male reproductive function, possibly by disturbing testosterone levels, and CPY11A1 may be a major steroidogenic enzyme targeted by PFOA.	[19]
PFOA	Adult exposure in vitro exposure of Sertoli cells	Male mice: 0–20 mg/kg/day by oral gavage for 28 days Sertoli cells: 0–500 μM for 48 h	BALB/c male mice and Sertoli cells culture	Decreased pregnant females per male mouse, decreased litter weight Damaged blood–testis barrier Decreased levels of claudin-11, connexin-43, *N*-cadherin, *β*-catenin, and occludin in the testes	Sertoli cells appear to be target of PFOA and the disruption of the blood–testis barrier may be crucial for PFOA-induced reproductive dysfunction in mice.	[94]
PFOA	Gestational exposure	2.5 or 5 mg/kg PFOA daily by gavage during gestation	Kunming mice of Clean Grade	Decreased survival number of offspring at weaning Reduced testosterone in the male offspring Damage to testis in a dose-dependent manner Decreased number of Leydig cells	PFOA exposure during pregnancy reduces survival of offspring, damages the testis, and disrupts reproductive hormones.	[95]
PFOS	Prepubertal exposure	5 or 10 mg/kg PFOS on postnatal day 35 for 21 days	Sprague Dawley rats	Decreased testosterone levels Downregulated expression of *Lhcgr*, *Cyp11a1*, and *Cyp17a1* in Leydig cells Inhibited androgen secretion in immature Leydig cells Increased apoptosis in Leydig cells	PFOS directly inhibits pubertal development of Leydig cells.	[96]
PFOA	Adult exposure	Vehicle control or PFOA at 2.5 mg/kg (for Balb/c mice) and 7.5 mg/kg (for C57Bl/6 wild type and PPARα knockout mice) by oral gavage, once daily, 5 days per week for 4 weeks starting at 21 days of age	Balb/c, C57Bl/6 wild type mice, and C57Bl/6 PPARα knockout mice	Inhibited mammary gland growth in both Balb/c and C57Bl/6 wild type mice, but not in C57Bl/6 PPARα knockout mice Delayed or absence of vaginal opening and lack of estrous cycling during the experimental period Decreased ovarian steroid hormonal synthetic enzyme levels Reduced expression of estrogen- or progesterone-induced mammary growth factors	The effects of PFOA on the ovaries mediate its ability to inhibit mammary gland development in Balb/c and C57Bl/6 mice.	[97]
PFOA	Gestational exposure	2.5, 5 or 10 mg/kg/day of PFOA by gavage from gestational day 1 until the day of euthanasia	Kunming mice	Increased numbers of resorbed embryos Reduced serum progesterone levels Decreases in transcript levels for key steroidogenic enzymes Inhibited activities of superoxide dismutase and catalase Increased generation of hydrogen peroxide and malondialdehyde Down-regulated level of *Bcl-2* Up-regulated p53 and BAX proteins	PFOA exposure significantly inhibits luteal function via oxidative stress and apoptosis in pregnant mice.	[98]
PFOA	in vitro exposure of oocytes and Ex vivo exposure of fetal ovaries	in vitro oocytes: 50, 100, and 150 μM for 24 h Ex vivo fetal ovaries: 28.2 μM	CD-1- mice oocytes CD-1- mice fetal ovarian tissue	Induced oocyte apoptosis and necrosis in vitro Increased ROS Caused the blockage of GJIC in cumulus cells-oocyte complexes	The ability of PFOA to disrupt the GJIC in COCs, generate ROS in the fetal ovary, and cause apoptosis and necrosis in oocytes might account for the reported association between increasing maternal plasma concentrations of PFOA with reduced fertility in women.	[99]
PFOS, PFOA, and perfluorohexane sulfonic acid	Adult exposure	PFOS, PFOA, and perfluorohexane sulfonic acid (medians of 24.5, 4.9, and 6.6 ng/mL, respectively)	105 Danish men from the general population (median age, 19 years)	Men with high combined levels of PFOS and PFOA had a median of 6.2 million normal spermatozoa in their ejaculate in contrast to 15.5 million among men with low PFOS-PFOA	High PFAS levels were associated with fewer normal sperm. High levels of PFAS may contribute to the otherwise unexplained low semen quality often seen in young men.	[100]
PFOA, PFOS	Gestational exposure	Range of maternal serum concentrations of selected PFAS: 1.26–54.28 ng/mL	169 male offspring (19–21 years of age) from a pregnancy cohort established in Aarhus, Denmark, in 19881989–	in utero exposure to PFOA was associated with lower adjusted sperm concentration and total sperm count and with higher adjusted levels of luteinizing hormone and follicle-stimulating hormone	in utero exposure to PFOA may affect adult human male semen quality and reproductive hormone levels.	[101]
PFOA	in vitro human semen exposure	PFOA 0.25, 2.5 or 25 μg/mL alone or in combination with progesterone	Mature human sperm	Reduced capacity of human spermatozoa to penetrate synthetic mucus Increased production of reactive oxygen species Compromised progesterone-induced acrosome reaction and sperm penetration into viscous medium	PFOA exposure may impair human sperm function through inducing oxidative stress and disturbing progesterone-induced Ca^2+^ signaling.	[102]
Perfluorodecanoic acid, perfluorohexane sulfonic acid, perfluorononanoic acid, PFOA, PFOS, perfluorododecanoic acid, perfluoroheptanoic acid, perfluorooctanesulfonamide, and perfluoroundecanoic acid measured in serum	Adult exposure	Range of serum perfluorochemical concentration: 0–43.2229 ng/mL	Operative sample: 495 women aged 18–44 years from clinical sites in the Salt Lake City or San Francisco area, US 2007–2009 Second sample: 131 women that matched to the operative sample on age and residence within a 50-mile radius of participating clinics	Serum PFOA and perfluorononanoic acid were associated with endometriosis in the operative sample Perfluorooctane sulfonic acid and PFOA increased the odds for moderate/severe endometriosis	Select PFAS are associated with endometriosis diagnosis.	[103]
PFOA, PFOS, perfluorohexane sulfonic acid, 2-(N-ethyl-PFOSA) acetate, 2-(N-methyl-PFOSA) acetate, perfluorodecanoic acid, perfluorobutane sulfonate, perfluoroheptanoic acid, perfluorononanoic acid (PFNA), perfluorooctane sulfonamide, perfluoroundecanoic acid, and perfluorododecanoic acid measured in serum	Adult exposure	Range of serum concentratios of selected PFAS: 0.07–392 ng/mL	753 women aged 20–50 years from the National Health and Nutrition Examination Survey (2003–2006) in US	Geometric mean levels of perfluorononanoic acid, PFOA, and PFOS were higher among women reporting endometriosis and endometriosis was associated with select quartiles of PFOA, PFNA, and PFOS	PFOA, PFNA, and PFOS may be associated with an increased risk of endometriosis.	[104]
Perfluorododecanoic acid, perfluoroundecanoic acid, perfluorodecanoic acid, perfluorooctane sulfonamide, PFOS, PFOA, perfluoroheptanoic acid, perfluorohexane sulfonic acid, perfluorobutane sulfonic acid (PFBS) measured in plasma	Adult exposure	Range of plasma concentratios of selected PFAS: 0.006–138 ng/mL	157 Chinese women aged 20–45 surgically confirmed endometriosis cases and 178 seeking infertility treatment because of male reproductive dysfunction in 2014 and 2015	Plasma concentrations of PFBS were associated with an increased risk of endometriosis-related infertility	Exposure to PFBS may increase the risk of female infertility due to endometriosis.	[105]
PFOA, PFOS perfluorohexane sulfonic acid, 2-(N-ethyl-PFOSA) acetate (EPAH), 2-(N-methyl-PFOSA) acetate, perfluorodecanoic acid, perfluorobutane sulfonate, perfluoroheptanoic acid, perfluorononanoic acid (PFNA), perfluorooctane sulfonamide, perfluoroundecanoic acid, perfluorooctanesulfonic, and perfluorododecanoic acid measured in blood	Adult exposure	N/A	178 healthy, naturally cycling women, aged 25–35 years in Tromsø, Norway	PFOS blood concentrations were inversely associated with salivary concentration of estradiol and progesterone Similar, but weaker results were observed for PFOA	PFOS and perfluorooctanesulfonic acid may be associated with decreased production of estradiol and progesterone in reproductive-age women.	[106]
Perfluorohexanesulfonate (PFHxS), perfluoroheptanoic acid (PFHpA), perfluorononanoic acid (PFNA), perfluorooctanoic acid (PFOA), perfluorooctyl sulfonate (PFOS), perfluorodecanoic acid (PFDeA), perfluoroundecanoic acid (PFUA), perfluorododecanoic acid (PFDoA), 2-(N-methyl-perfluorooctane sulfonamido) acetic acid (Me–PFOSA–AcOH), 2-(N-ethylperfluorooctane sulfonamido) acetic acid (Et–PFOSA–AcOH), perfluorohexanoic acid (PFHxA), and perfluorooctane sulfonamide (PFOSA) measured in serum	Adult exposure	Range of serum concentratios of selected PFAS: 3.63–13.41 ng/mL	540 subjects aged 12–30 years from a 1992 to 2000 in Taiwan	The adjusted mean serum level of sex hormone-binding globulin decreased in association with PFOA blood concentration Follicle-stimulating hormone levels were decreased in association with PFOS in the male 1217–-year-old group and with perfluoroundecanoic acid (PFUA) in the female 1217–-year-old group	Serum concentrations of PFOA, PFOS, and PFUA were negatively associated with the serum levels of sex hormone-binding globulin, follicle-stimulating hormone, and testosterone in young Taiwanese population and these effects were the strongest in the females aged 12–17.	[107]
PFOA, PFOS measured in plasma	Gestational exposure	PFOS and PFOA levels in maternal plasma were on average 35.3 and 5.6 ng/mL, respectively	1,400 women and their infants from the Danish National Birth Cohort	PFOA levels were inversely associated with birth weight	Maternal plasma PFOA levels are inversely associated with birth weight.	[108]
PFOS, PFOA, and perfluorohexane sulfonate (PFHxS) measured in serum	Maternal exposure	Range of serum concentratios of selected PFAS: 0.1–36 ng/mL	The Maternal-Infant Research on Environmental Chemicals Study is a cohort study of 2,001 women recruited before 14 weeks of gestation in 10 cities across Canada between 2008 and 2011	PFOA and PFHxS were associated with a 11 and 9% reduction in fecundability The odds of infertility increased by 31% per one standard deviation increase of PFOA	Exposure to PFOA and PFHxS, even at lower levels than previously reported, may reduce fecundability.	[109]
PFHxS, PFOS, PFOA, PFNA, perfluorodecanoic acid (PFDA), perfluorodecane sulfonate, perfluoroundecanoic acid, perfluorododecanoic acid, perfluorotridecanoic acid, perfluorotetradecanoic acid, and perfluorohexadecanoic acid measured in maternal plasma	Maternal exposure	Range of plasma concentratios of selected PFAS: 0.01–500 ng/mL	1292 pregnant women in Shanghai, China, 2012	Maternal plasma concentrations of PFOS, PFDA, and perfluoroundecanoic acid were inversely associated with anogenital distance at birth in male offspring	Higher maternal concentrations of some PFAS during pregnancy are associated with shorter anogenital distance in male infants.	[110]
PFOA	Adult exposure and in vitro exposure of Ishikawa cells	N/A	146 exposed females aged 18–21 from the Veneto region in Italy and 1080 non-exposed controls andhuman endometrial Ishikawa cells	Dysregulation of the genetic cascade leading to embryo implantation and endometrial receptivity Molecular interference with progesterone Increased age at menarche (+164 days, *p*=0.006) and frequency of girls with irregular periods	PFAS have endocrine-disrupting activity on progesterone-mediated endometrial function.	[111]
PFOA and PFOS	Adult exposure nd in vitro exposure of HeLa cells	Range of serum concentratios of PFOA and PFOS: 0–156.7 ng/mL in vitro exposure: 1 µM PFOA or PFOS	212 exposed males and 171 non-exposed males controls in Veneto Region, Italy from 2017 to 2018	Reduced semen quality, testicular volume, penile length, and anogenital distance Antagonistic effect of PFOA on testosterone and androgen receptor binding	PFOA and PFOS exposure affects androgenic function and impairs reproductive outcomes in males.	[112]

**Table 5:** Effects of BPA on the Reproductive System.

Chemical	Exposure Window	Dose	Model/Study Population	Effects	Conclusion	Reference
BPA	Adult exposure	Oral administration of corn oil or 20 μg/kg of BPA Injection of 0, 0.02, 0.2, 2.0, 20.0, and 200.0 nM of BPA into the right lateral ventricle	ICR female mice	Oral administration of BPA at proestrus increased the levels of plasma estradiol, LH and FSH, and *Gnrh* mRNA Oral administration of BPA at proestrus elevated the levels of *Kiss1* mRNA and kisspeptin protein in anteroventral periventricular nucleus (AVPV) At proestrus, a single injection of BPA enhanced AVPV-kisspeptin expression and elevated the levels of plasma E_2_, LH, and *Gnrh* mRNA	Exposure of adult female mice to a low dose of BPA disrupts the hypothalamic–pituitary–gonadal reproductive endocrine system through enhancing AVPV-kisspeptin expression and release.	[139]
BPA	Gestational exposure	0, 8, 40 and 200 mg/kg by gavage from gestational day 0 to 18	CD-1 mice	Accelerated vaginal opening Altered sex hormones levels	Maternal exposure to BPA resulted in advancing puberty and increased GnRH hormone levels, affecting the function of the HPG axis in female offspring.	[140]
BPA	Gestational exposure	Dimethylsulfoxide vehicle-treated, 25 μg/kg, and 250 μg/kg (subcutaneous injections)	Wistar rats	Enlarged layer of fibroblasts in the prostatic periductal stroma Increased cellular proliferation in the stroma Decreased expression of androgen receptor in prostatic stromal cells and prostatic acid phosphatase in epithelial cells	Prenatal exposure to environmental doses of BPA induced both transient and permanent age-dependent alterations in the male reproductive axis at different levels.	[141]
BPA	Gestational exposure	Corn oil, 25 mg BPA/kg/day, or 50 mg BPA/kg/day by gavage	Six-week-old male and female CD-1 mice	Up-regulated *Kiss1*, *GnRH* and *Fsh* mRNA in both male and female pups Inhibited expression of testicular steroidogenic enzymes and synthesis of testosterone in the male pups Increased aromatase expression and synthesis of estrogen in the female pups	The effects of BPA on reproductive dysfunction may be due to its actions on gonadal steroidogenesis and on the anomalous releases of endogenous steroid hormones.	[142]
BPA	Adult exposure	5 or 25 mg BPA/kg/day	Wistar rats	Reduced sperm production, reserves, and transit time Increased levels of defective spermatozoa Increased expression *Gnrhr*, *Lhb*, *Fshb*, *Esr2*, and *Ar* in the pituitary and reduced expression of *Esr1* in the hypothalamus Reduced serum concentrations of testosterone, LH and FSH and increased concentration of estradiol	At dosages previously considered nontoxic to reproductive function, BPA compromises spermatozoa and disrupts the hypothalamic-pituitary-gonadal axis, causing a state of hypogonadotropic hypogonadism.	[143]
BPA	Adult exposure	Control, 1, 10, 100 and 1000 µg/L	Zebra fish	Decreased and increased expression of *Cyp19b* depending on the dose	BPA dysregulates gonadotropic hormones, causing degeneration of gonadotropic cells.	[144]
BPA	Neonatal exposure	Daily subcutaneous injections of 0.5 or 50 μg BPA, 50 μg BPA plus 100 IU retinol acetate or the vehicle only, for 5 days from the day of birth.	SHN mice	Decreased percentage of moving sperm, Increased incidence of malformed sperm The deteriorating effects of 50 µg of BPA were ameliorated by the concurrent administration of 100 IU of retinol acetate	Neonatal exposure to a relatively large dose of BPA causes damage to the motility and morphology of sperm, but the BPA effect is, to some extent, inhibited by a supplement of retinol acetate, and enhanced under a retinol acetate deficiency condition.	[145]
BPA	Pubertal exposure	BPA (5, 10, and 20 mg/kg), X-rays (0.05 Gy), or a combination of both (0.05 Gy + 5 mg/kg BPA)	Pzh:Sfis mice	BPA and X-rays alone diminished sperm quality BPA exposure significantly reduced sperm count in pubescent males compared to adult mice, with degenerative changes detected in seminiferous epithelium Increased vacuolization of Sertoli cells and spermatogonia in animals treated with BPA and X-rays	Combined BPA with X-ray treatment enhanced the harmful effect induced by BPA alone in male germ cells of adult males, whereas low-dose irradiation showed sometimes protective or additive effects in pubescent mice.	[146]
BPA	Gestational exposure	Water (negative control), olive Oil (vehicle control), diethylstilbestrol (DES-positive control-6.5 μg/kg, and BPA (40, 80 and 200 μg/kg)	Vesper mice	BPA reduced normal sperm morphology, sperm membrane integrity, sperm motility, and in vitro penetration rates	in utero exposure to BPA caused a reduction in sperm parameters of adult *C. laucha*.	[147]
BPA	Adult exposure	0, 10, 50, and 250 μg/kg were administrated orally to for 8 weeksor applied directly to normal mouse sperm in vitro	C57BL/6 mice	Decreased sperm motility and acrosome reaction in BPA treated mice Inhibited CatSper transiently and reduced sperm total motility and acrosome reaction ratio in vitro	Both in vivo administration and in vitro application of BPA impaired mature sperm function by a CatSper-relevant mechanism.	[148]
BPA	Neonatal exposure	Blank control group, negative control group (corn oil) and BPA 100 μg/kg group.	ICR mice	Decreased diameter and the epithelium thickness of seminiferous epithelium Increased lumen in the seminiferous tubules Decreased expression and protein level of Boule mRNA	Neonatal BPA exposure has a long-term effect on mouse testicular development and may affect testicular development by decreasing the expression of Boule mRNA and protein in testes.	[149]
BPA	Gestational exposure	Pregnant mice were injected intraperitoneally daily with sesame oil vehicle control or 25 μg/kg BPA	CD-1 mice	Increased defects in the developing mammary epithelium Altered *Ki67* and *ERα* expression	BPA exposure caused increased defects in the developing mammary epithelium and altered *Ki67* and *ERα* expression.	[150]
BPA	Adult exposure	Control and experimental groups (5 mg/kg BPA daily for 8 weeks)	Albino rats	Increased number and size of the acini and ducts in the mammary gland of treated rats with hyperplasia of their lining epithelial cells Increased collagen content in the connective tissue stroma separating the ducts Increased Ki67 and caspase-3 levels	BPA induced structural changes and affected the proliferation rate of mammary glands.	[151]
BPA	Adult exposure	Experiment 1: BPA at doses of 0, 1 mg, 10 mg, 100 mg, 1 mg, and 10 mg/kg every day, respectively, for 28 days Experiment 2: oral BPA at doses of 0, 1 mg, and 10 mg/kgExperiment 3: oral BPA at doses of 0, 100 mg, and 10 mg/kg	CD-1 mice	Downregulaion of *PTEN* expression Accelerated premature activation of primordial follicles and this effect was partly reversible by *PTEN* overexpression	BPA initiates excessive premature activation of primordial follicles in themature mouse ovaries via the PTEN/PI3K /AKT signaling pathway.	[152]
BPA	Perinatal exposure	BPA 0.05 mg/kg, 20 mg/kg, or vehicle, from gestational day 6 to lactation day 21	Wistar rats	Induced alterations in progesterone and estradiol serum levels, and implantation rate Altered levels of claudin-1, claudins -3, claudins -4, and claudins -7 in stromal cells Altered levels of ZO-1 in stromal cells	BPA treatment during the perinatal period perturbs the expression of tight junction proteins in the uterine epithelium and reduces the number of implantation sites.	[153]
BPA	Adult exposure	Oral BPA 60 µg/kg dissolved in ethanol, and suspended in tocopherol-stripped corn oil for 0.1% final ethanol concentration or vehicle tocopherol-stripped corn oil + 0.1% ethanol	C57BL6 mice	Increased proliferation, of the glandular epithelium Reduced expression of heart and neural crest derivatives expressed 2 (HAND2) Increased methylation of a CpG island in the *Hand2* gene promoter	Chronic oral exposure to a low concentration of BPA during adulthood impedes transcriptional activation of the antiproliferative factor HAND2, likely through an epigenetic mechanism involving hypermethylation at the *Hand2* promoter.	[154]
BPA	Adult exposure	Control, 0.05 mg/kg/day of BPA, or 0.5 mg/kg/day of BPA	Piétrain × Duroc sows	Increased number of NRG-1-LI positive nerves in the uterus Increased changes in neurochemical characterization of NRG-1-LI nerves in the uterine wall	NRG-1 in nerves supplying the uterus may play roles in adaptive and protective mechanisms under the impact of BPA.	[155]
BPA	Adult exposure	Median serum BPA concentrations 0.19 vs. 0.18 ng/mL in healthy and infertile men, respectively	55 infertile men, in Greece	High concentrations of BPA (>3 ng/ml) were observed only in infertile men A negative correlation was observed between ΒΡA concentrations and AMH	Very high concentrations of BPA are associated with azoospermia.	[156]
BPA	Adult exposure	Range of urinary BPA concentrations from 0.16–11.5 ng/mL	215 healthy young male students (18–23 years old), investigated between 2010 and 2011 in Southern Spain	Positive association between urinary BPA concentrations and serum LH levels Urinary BPA concentration inversely associated with sperm concentration	BPA exposure may be associated with a reduction in Leydig cell capacity (increased LH levels) and decreased sperm counts in young men.	[5]
BPA	Adult exposure	Medians of unadjusted BPA concentrations: 0.32 (0.08–6.86) μg/L	500 men aged 18–55 years and having at least one child, in Guizhou Province, China, 2012	Subjects in the highest tertile of creatinine-adjusted BPA group had lower sperm concentration than those with undetected BPA	Exposure to environmental BPA decreases sperm concentration and sperm swing characteristics (ALH and MAD), and increases sperm velocity ratios (LIN, STR and WOB), which might mediate further effects on impaired male fecundity.	[157]
BPA	Adult exposure	BPA median concentrations of infertile patients and fertile controls: 24.2 μg/L and 20.9 μg/L, respectively	50 infertile patients and 50 matched controls in Upper Egypt	Total BPA levels were negatively associated with semen quality and antioxidant levels Total BPA levels were positively correlated with DNA damage	BPA levels showed stronger associations with semen quality parameters, sperm DNA integrity and oxidative stress in infertile than fertile men sampled from Upper Egypt.	[158]
BPA	Adult exposure	BPA concentrations ranged from <0.4 to 25.5 µg/l	315 men under 45 years of age with normal sperm concentration in Poland	Positive association between the urinary concentrations of BPA 25th–50th percentile and total sperm sex chromosome disomy Urinary concentration of BPA associated with increased total sperm sex chromosome disomy Urinary concentration of BPA associated with increased percentage of immature sperm and decreased motility	Exposure to BPA is associated with poor semen quality.	[159]
BPA	Adult exposure	Urinary BPA concentrations ranged from <0.4 to 25.5 µg/l	84 women (mean age 35.6 years) undergoing 112 IVF cycle in Massachusetts, US	Urinary BPA concentrations were inversely associated with the number of oocytes retrieved and peak estradiol levels	BPA was detected in the majority of women undergoing IVF, and BPA urinary concentrations were found to be inversely associated with the number of oocytes retrieved per cycle and peak serum estradiol levels.	[160]
BPA	Pubertal exposure	BPA urinary levels peripheral precocious puberty 8.7 ± 7.6 μg/g creatinine; central precocious puberty 8.0 ± 9.9 μg/g creatinine	32 healthy girls (age, 8.5 ± 0.9 years), 40 girls with peripheral precocious puberty (age, 8.4 ± 0.7 years), and 42 girls with central precocious puberty (age, 8.7 ± 1.0 years) in Korea	High urinary BPA levels were associated with increased levels of testosterone, 17β-estradiol, and pregnenolone	In girls, BPA exposure is associated with metabolic changes in steroidogenesis, but not the early onset of precocious puberty.	[161]
BPA	Adult exposure	The median urinary concentration of BPA was 1.29 ng/mL (interquartile range: 0.69 ng/mL–2.34 ng/mL)	700 women attempting pregnancy and followed for 12months or until a pregnancy occurred in China from 2013 to 2015	Urinary concentrations of BPA were associated with a 13% reduction in fecundability and a 23% increase in odds of infertility Women in the highest quartile of urinary BPA had a 30% reduction in fecundability and a 64% increase in odds of infertility when compared to those in the lowest quartile	Preconception concentrations of BPA in female urine were associated with decreased fecundability, particularly among women at older ages.	[162]
BPA	Maternal exposure	Range of BPA serum levels in patients with miscarriage patients was 0.0419–4.7900 and in patients with live birth cases was 0.0020–1.4390 ng/mL	115 women included in the study, there were 47 live births and 68 clinical miscarriages in California, US	Median conjugated BPA concentrations were higher in the women who had miscarriages than in those who had live births Women with the highest quartile of conjugated BPA had an increased relative risk of miscarriage compared with the women in the lowest quartile	Maternal conjugated BPA is associated with a higher risk of aneuploid and euploid miscarriage.	[163]
BPA	Maternal exposure	Serum BPA levels in patients were 2.59 ± 5.23 ng/mL and 0.77 ± 0.38 ng/mL for control women	45 patients with a history of three or more consecutive first-trimester miscarriages and 32 healthy women with no history of live birth and infertility inNagoya City, Japan between August 2001 and December 2002	High exposure to bisphenol A was associated with the presence of antinuclear antibodies	Exposure to bisphenol A is associated with recurrent miscarriage.	[164]
BPA	Maternal exposure	Levels of BPA analytes in maternal and umbilical cord plasma ranged from 0.05–34.24 ng/mL	80 pregnant womenin Michigan, US	A two-fold increase in first trimester maternal BPA was associated with 55 g less birth weight when male and female pregnancies were combined and 183 g less birth weight with only female pregnancies A two-fold increase in maternal term BPA was associated with an increased gestational length of 0.7 days for all pregnancies and 1.1 days for only female pregnancies	Higher BPA exposure levels during first trimester and term are associated with sex-specific reduction in birth weight and increase in gestational length, respectively.	[165]
BPA	Maternal exposure	Amniotic fluid BPA concentrations ranged from ≤0.25 ng/mL to >2.0 ng/mL	Amniotic fluid samples were collected from women with ages ranging from 16 to 45 years that had healthy singleton pregnancies with infants born at term in Philadelphia, US	The mean body weight of infants with amniotic fluid BPA 0.40–2.0 ng/mL was 241.8 g less than infants with amniotic fluid BPA less than the limit of quantification after controlling for covariates	Low level BPA exposure in utero decreases body weight.	[166]
BPA	Maternal exposure	Concentration of bisphenols and phthalates in urine ranged from 0.33–1080.01 ng/mL	1213 pregnant women in Rotterdam, the Netherlands	Higher total bisphenols and bisphenol S were associated with lower total gestational weight gain specifically in normal weight women Total bisphenol and BPA urine concentrations were associated with lower mid- to late pregnancy gestational weight gain	Higher maternal bisphenol urine concentrations in early pregnancy may lead to reduced gestational weight in the second half of pregnancy.	[167]
BPA	Maternal exposure	The median of the unadjusted urinary BPA was 4.70 μg/L in low birth weight infants cases, and 2.25 μg/L in the controls	452 mother-infant pairs in Wuhan city, China, during 2012–2014	Mothers with low birth weight infants had significantly higher urinary BPA levels than the control mothers Increased risk of low birth weight was associated with higher maternal urinary levels of BPA The association was more pronounced among female infants than among male infants	Prenatal exposure to higher levels of BPA may potentially increase the risk of delivering low birth weight infants, especially for female infants.	[168]

**Table 6:** Effects of Phthalates on the Reproductive System.

Chemical	Exposure Window	Dose	Model/Study Population	Effects	Conclusion	Reference
DEHP	Gestational exposure	20 μg/kg/day 200 μg/kg/day 500mg/kg/day	CD-1 mice	Impaired testosterone production, reduced sperm quality, and decreased fertility	Prenatal phthalate exposure has adverse reproductive outcomes in male mice.	[6]
DBP	Gestational exposure	500 mg/kg/day	Sprague-Dawley rats F1, F2, and F3 generations	Decreased numbers of sperm and Sertoli cells	Prenatal phthalate exposure has adverse reproductive outcomes in male mice.	[182]
DEHP	Gestational exposure	500 mg/kg/day	CD-1 mice F3 and F4 generations	Abnormal seminiferous tubules	Prenatal phthalate exposure has adverse reproductive outcomes in male mice.	[183]
DEHP	Adult exposure	1–100 ml/kg	Male and female mice 8–10 weeks of age	Females: Reduced incidence of pregnancy, damage to ovarian germ cells, fewer and smaller corpora lutea Males: Reduced testicular weight, atrophy of seminiferous tubules, chronic inflammation of testes Both: elevated activity of lysosomal enzymes	Prenatal phthalate exposure has adverse reproductive outcomes in male mice.	[184]
MEHP	Gestational exposure	100 mg/kg 500 mg/kg 1000 mg/kg	Female C57/Bl6 mice	Decreased reproductive lifespan, delayed estrous onset, prolonged estrus, elevated serum FSH and estradiol, altered mRNA expression of steroidogenic enzymes	Prenatal phthalate exposure has adverse reproductive outcomes in male mice.	[185]
DEHP	Gestational exposure	0.05 mg/kg/day 5 mg/kg/day	Female mice	Reduced oocyte quality, reduced embryonic developmental competence Dysregulation of genes responsible for ovarian and embryonic development	In utero exposure to DEHP has adverse reproductive outcomes.	[186]
DEHP	Gestational exposure	1 mg/kg/day 20 mg/kg/day 50 mg/kg/day 300 mg/kg/day Gestational day 14 until birth	Female mice F1, F2, F3	F1: Reduced estrogen levels at proestrous and estrous, decreased thecal cell layers F3: Decreased weights, rates of pregnancy and increased litter size	The adverse reproductive outcomes of DEHP are transgenerational.	[187]
DEHP	Gestational exposure	20 μg/kg/day 200 μg/kg/day 500 μg/kg/day 750 mg/kg/day Gestational day 10.5 until birth	Female CD-1 mice	F1: decreased rate of pregnancy and fertility indices F3: female biased litters, decreased anogenital distance	The adverse reproductive outcomes of DEHP are transgenerational.	[188]
DEHP	Postnatal exposure	20 μg/kg/day 40 μg/kg/day	Female CD-1 mice	Decreased expression of steroidogenic genes, reduced ovarian concentrations of progesterone, estradiol, and androstenedione, reduced LH in serum, altered structure of theca cells	DEHP exposure shortly after birth has adverse reproductive outcomes.	[189]
Urinary metabolites of DiNP	Gestational exposure	average 67.74 ng/ml	197 infant males from Sweden, 21 months of age	Reduced anogenital distance	DiNP is not a suitable replacement for DEHP because it is associated with adverse reproductive outcomes.	[190]
Urinary metabolites of DEHP	Gestational exposure	1.93–71.7 ng/ml	366 boys from US examined shortly after birth	Reduced anogenital distance	Environmental exposure to DEHP is associated with adverse male genital development.	[191]
Urinary phthalate metabolites	Adult exposure	0.54–18.5 ng/ml	501 adult males in US	Lower total sperm counts and concentrations, larger sperm head sizes, lower and higher sperm motility	Phthalate exposure is associated with adverse reproductive outcomes in adult males.	[192]
Serum DEHP and DiNP metabolites	Adult exposure	0.01–1.7 ng/ml	589 adult males from Greenland, Poland, and Ukraine	Decreased testosterone, decreased semen volume and total sperm count	Consistent findings of the anti-adrenergic effects of phthalates.	[193]
DEHP and DiNP in maternal sera	Gestational exposure	0.026–5.2 ng/ml	112 adolescent males in Sweden	DiNP: Decreased testicular volume and semen volume, higher levels of follicle stimulating hormone DEHP: decreased semen volume	DEHP and DiNP exposure is associated with adverse reproductive outcomes in adolescent males.	[194]
Urinary DEHP, DBzP, and DBP metabolites	Adult exposure	4.5–83.4μg/l	269 men in US	Decreased sperm motility, decreased testosterone levels, increased sperm DNA damage and sperm aneuploidy	Phthalate exposure is associated with adverse reproductive outcomes in adult males.	[195]
Urinary DiBP, DEHP, DBP, and DBzP metabolites	Adult exposure	0.2–78.7 ng/ml	1066 adult Chinese men	Decreased serum testosterone and luteinizing hormone Decreased Leydig cell production of INSL3	Phthalates are associated with disrupted steroidogenesis in adult males.	[196]
Urinary DBP metabolites	Adult exposure	Average 17–7 ng/ml	463 adult males in US	Decreased sperm concentration and motility	DBP is associated with altered semen quality in adult males.	[197]
Urinary metabolites of DBzP	Adult exposure	Average 11.13 ng/ml	420 adult males in US	Decreased sperm concentration and motility	DbzP alters semen quality on adult males.	[198]
Urinary metabolites of DEHP and DBzP	Adult exposure	0.9–5.2 ng/ml	176 Taiwanese adult males	Decreased serum testosterone, serum inhibin B, and Leydig cell production of INSL3	Non-occupational exposure to DEHP and DBzP is associated with adverse effects on testicular and Leydig cell function.	[199]
Maternal urine concentrations of DBzP and DEP	Gestational exposure	4.1–114 μg/l	116 adolescent females in Mexic	Increased testosterone levels	in utero phthalate exposure is associated with increased testosterone levels in adolescent females.	[200]
Maternal DEHP urine concentrations	Gestational exposure	0.04–0.08μg/l	131 children 8 and 11 years old in Taiwan	Reduced uterus size	Phthalate exposure is associated with negative pubertal development characteristics in female children.	[201]
Urine metabolites	Adult exposure	2.5–56.2 ng/ml	195 midlife women 45–54 years of age in US	Increases risk and frequency of hot flashes	Phthalate exposure is associated with adverse health outcomes in midlife women.	[202]

**Table 7:** Effects of Pesticides on the Reproductive System.

Chemical	Exposure Window	Dose	Model/Study Population	Effects	Conclusion	Reference
Atrazine	30 days	38.5 mg/kg/day 77 mg/kg/day 154 mg/kg/day	Sprague-Dawley male rats	Irregular and disordered arrangement of seminiferous epithelium Decreased spermatozoa number, increased spermatozoa abnormalities Decreased serum testosterone and inhibin B, increased serum FSH and LH	Atrazine exposure results in adverse reproductive outcomes in male rats.	[238]
Atrazine	Postnatal day 22 to 41	12.5 mg/kg/day 25 mg/kg/day 50 mg/kg/day 100 mg/kg/day 200 mg/kg/day	Wistar female rats	Delayed onset of puberty and altered estrous cyclicity	Postnatal atrazine exposure delays onset of puberty in female rats.	[239]
Simazine	Maternal	5–500 µg/kg	Female offspring mice	Shortened anogenital distance Decreased ovarian and uterine weights Increased apoptotic granulosa cells	Maternal exposure to simazine impairs reproductive development of female offspring.	[241]
Simazine	21 days and 41 days starting on post-natal day 22	12.5 mg/kg/day 25 mg/kg/day 50 mg/kg/day 100 mg/kg/day	Wistar female rats	Delayed vaginal opening, decreased number of cycles, day of first estrus delayed, decreased serum prolactin	Simazine delays onset of puberty.	[242]
Simazine	Maternal during gestation and lactation	5 µg/kg 50 µg/kg 500 µg/kg	Male offspring mice	Decreased testicular and epididymal weight Increased testicular apoptosis Decreased sperm concentrations	Maternal exposure to simazine has adverse reproductive outcomes in male offspring.	[243]
Metolachlor	Postnatal day 23 to 53	5 mg/kg/day 50 mg/kg/day	Wistar male rats	Increased serum testosterone, estradiol and FSH Higher amount of fluid in seminal vesicles, precocious puberty, and changes in seminiferous epithelial morphology	Prepubertal exposure to metolachlor is associated with adverse reproductive outcomes in male rats.	[244]
Acetochlor	30 days	250 mg/kg/day 500 mg/kg/day 1000 mg/kg/day	Adult C57/BL6 male mice	Increased markers of oxidative stress, degeneration of testicular tissue Elevated apoptosis, elevated expression of apoptotic proteins	Acetochlor can induce reproductive toxicity in subacutely exposed mice.	[245]
Acetochlor	Postnatal day 4 to 7	7.68 mg/kg/day 15.36 mg/kg/day	Wistar female rats	Acceleration of vaginal patency, irregular cycled, accumulation of uterine nuclear estrogen receptors	Neonatal exposure to acetochlor alters pubertal development in female rats.	[246]
Malathion	50 days	33.75 mg/kg 54 mg/kg 108 mg/kg	Wistar male rats	Lower testis weights, sperm motility, higher sperm malformation rates Higher spermatogenic cell apoptosis rates, higher Bax expression, lower *Bcl-2* expression, damage to seminiferous tubules	Exposure to malathion has a negative effect on reproductive health of male rats.	[247]
Chlorpyriphos	45 days	37 mg/kg/day	Male rats	Deceases in sperm counts, viability, and motility, increased sperm DNA damage, Increased arrested spermatogenesis, decreased Leydig cell number, negative tubular differentiation and repopulation indices	Exposure to chlorpyriphos has a negative effect on the reproductive health of male rats.	[248]
Chlorpyriphos	90 days	2.7 mg/kg 5.4 mg/kg 12.8 mg/kg	Male rats	Reduced testicular sperm counts, sperm motility, increased rates of sperm malformation Degenerative changes in seminiferous tubules, increased FSH, decreased testosterone,	Chlorpyriphos has adverse effects on the male reproductive system.	[249]
Imidacloprid	90 days	0.5 mg/kg/day 2 mg/kg/day 8 mg/kg/day	Male rats	Decreased epidydimal weight and sperm concentration Higher rates of apoptosis and DNA fragmentation, and abnormal sperm	Imidacloprid has a negative effect on the male rat reproductive system.	[250]
Imidacloprid	90 days	5 mg/kg/day 10 mg/kg/day 20 mg/kg/day	Female rats	Decreased ovarian weight, pathomorphological changes in follicles Alterations in levels of LH, FSH, and progesterone	Imidacloprid is toxic to the ovary in female rats.	[251]
Clothianidin	90 days	2 mg/kg/day 8 mg/kg/day 24 mg/kg/day	Male rats	Decreased epidydimal weight, and weights of seminal vesicle, Elevated palmitic, linoleic, and arachidonic acids in testes	Clothianidin has a negative effect on the male rat reproductive system.	[252]
Atrazine and	Lifetime	Missouri: 0.17 mg/l Minnesota: 0.07 mg/l	86 men from Missouri and Minnesota, US	Reduced sperm count	Atrazine exposure in men can reduce their sperm count.	[253]
Atrazine and Metolachlor	Intrauterine	2.2 mg/l	Women living in 13 communities served by the Rathbun water system in Iowa, US	Intrauterine growth retardation	Women with high atrazine exposure are susceptible to intrauterine growth retardation.	[254]
Malathion and diazinon	Exposure during first trimester of pregnancy		Women at approximately 22 weeks of gestation in Sicily, Italy	Higher incidence of gestational hypertension	Women with organophosphorus pesticide exposure in their first trimester of pregnancy have greater incidence of gestational hypertension.	[18]

**Table 8:** Effects of Environmental Estrogens on the Reproductive System.

Chemical	Exposure Window	Dose	Model/Study Population	Effects	Conclusion	Reference
17α-ethynylestradiol	Chronic exposure	5–6 ng/L	Fish fathead minnow (*Pimephales promelas*) in northwestern Ontario, Canada	Feminization of males through the production of vitellogenin mRNA and protein Impacted gonadal development as evidenced by intersex in males and altered oogenesis in females A near extinction of this species from the lake	Concentrations of estrogens and their mimics observed in freshwaters can impact the sustainability of wild fish populations.	[278]
N/A	Chronic exposure	N/A	Fish (Greenside Darters *Etheostoma blennioides* and Rainbow Darters *E. caeruleum*) in The Grand River watershed in Ontario, Canada	Impaired capacity to produce testosterone and 11-ketotestosterone in vitro, and in cellular development (GSI, intersex) in male fish Rates of intersex were elevated	Urban stretches of river are exposed to estrogenic compounds (man-made or otherwise), causing adverse biological effects (intersex in males) that might impair their ability to reproduce normally.	[279]
N/A	Chronic exposure	N/A	Wild fish sampled from rivers, lakes, or canals in British Isles, UK	High incidence of intersexuality in wild populations of riverine fish (roach; Rutilus rutilus)	Environmental estrogens my cause widespread sexual disruption in wild populations.	[280]
17β-estradiol, estrone, estriol, and 17α-ethynylestradiol,	Adult exposure	Estrone and 17β-estradiol concentrations ranged from <2.0 to >75 ng/L. Estriol concentrations 1.2 ± 0.7 ng/L and 17α-ethynylestradiol 3.4 ± 3.2 ng/l.	Adult male fathead minnows (Pimephales promelas). Study conducted in Colorado, US	Primary (sperm abundance) and secondary (nuptial tubercles and dorsal fat pads) sex characteristics were demasculinized Vitellogenin was maximally elevated	The reproductive disruption observed in this watershed is due to endocrine-active chemicals in the Colorado wastewater treatment plant effluent.	[281]
Estrone, 17β-estradiol, and diethylstilbestrol	Adult exposure	Maximum concentrations detected for estrone, 17β-estradiol, and diethylstilbestrol in surface waters were 32.0 ng/L, 3.7 ng/L and 22.0 ng/L, respectively.	Mosquitofish (*Gambusia affinis*) in rivers in South China	Induction of vitellogenin and ERα mRNA in the livers of the males and a gonopodium-like anal fin in the females collected at the majority of sites Chemical concentrations obtained by in vitro bioassays and chemical analysis had significant correlations with some of the endpoints for the estrogenic and/or androgenic effects in mosquitofish	Estrogens and androgens present in rivers could cause the observed estrogenic and androgenic effects in mosquitofish.	[283]
17α-ethinylestradiol	Developmental exposure	5 nM of 17α-ethinylestradiol.	Northern leopard frog (*Rana pipiens*)	Delayed in development immediately following exposure Tadpoles exposed early in development displayed a strong female-biased sex ratio compared to the controls.	Estrogen exposure can lead to significant delays in metamorphic development and result in feminized sex ratios both immediately following exposure and persisting through to metamorphic climax.	[284]
Ethinylestradiol and zearalenone	Adult exposure	Ethinylestradiol (10 μg/kg), zearalenone (10 mg/kg), or vehicle for 10 days starting from postnatal day 18.	Wistar rats	Both zearalenone and ethinylestradiol accelerated vaginal opening, increased the uterine weight and the number of antral follicles in the ovary, and resulted in increased central expression of *Gnrh* Increase of *Kiss1* mRNA in the anteroventral and rostral periventricular hypothalamus and an increased kisspeptin fiber density and kisspeptin -GnRH appositions in the preoptic area	Exposure of peripubertal rats to the structurally different xenoestrogens ethinyl estradiol and zearalenone advances puberty onset, very likely by increasing/amplifying the kisspeptinergic drive to GnRH neurons.	[285]
Daidzein, equol, genistein, naringenin, coumestrol, and secoisolariciresinol	Adult exposure	Phytoestrogens mean levels in urine ranged from 0.34–64.16 μg/g of creatinine.	608 idiopathic infertile men and 469 fertile controls in China	Exposures to daidzein, genistein and secoisolariciresinol were associated with idiopathic male infertility with abnormal sperm concentration, number per ejaculum and motility	Phytoestrogen exposures are related to male reproductive function and raise a public health concern because phytoestrogens exposure is ubiquitous in China.	[286]

## Abbreviations

EDCs	endocrine disruptor chemicals
USEPA	United States Environmental Protection Agency
BPA	bisphenol A
PFOA	perfluorooctanoic acid
PFOS	perfluorooctanesulfonic acid
DBPs	water disinfection byproducts
THMs	trihalomethanes
MCL	maximum contaminant level
HAAs	haloacetic acids
PFAS	perfluoroalkyl and polyfluoroalkyl substances
ROS	reactive oxygen species
USFDA	United States Food and Drug Administration
DEHP	di (2-ethylhexyl) phthalate
BBP	benzyl butyl phthalate
DiNP	di-isononyl phthalate
DnOP	di-n-octyl phthalate
DEP	diethyl phthalate
DMP	dimethyl phthalate
DBP	dibutyl phthalate
DiBP	diisobutyl phthalate
MMP	monomethyl phthalate
MEP	monoethyl phthalate
MiBP	monoisobutyl phthalate
MnBP	mono-n-butyl phthalate
MEHP	mono-2-ethylhexyl phthalate
PET	polyethylene terephthalate
PND	postnatal day
USGS	United States Geological Survey
DDT	dichlorodiphenyltrichloroethane
FSH	follicle stimulating hormone
LH	luteinizing hormone
